# Genetic investigation into the broad health implications of caffeine: evidence from phenome-wide, proteome-wide and metabolome-wide Mendelian randomization

**DOI:** 10.1186/s12916-024-03298-y

**Published:** 2024-02-20

**Authors:** Loukas Zagkos, Héléne T. Cronjé, Benjamin Woolf, Roxane de La Harpe, Stephen Burgess, Christos S. Mantzoros, Paul Elliott, Shuai Yuan, Susanna C. Larsson, Ioanna Tzoulaki, Dipender Gill

**Affiliations:** 1 Department of Epidemiology and Biostatistics, School of Public Health, Imperial College London, London, UK; 2 Department of Public Health, Section of Epidemiology, University of Copenhagen, Copenhagen, Denmark; 3 School of Psychological Science, University of Bristol, Bristol, UK; 4 Medical Research Council Integrative Epidemiology Unit, University of Bristol, Bristol, UK; 5 Medical Research Council Biostatistics Unit at the University of Cambridge, Cambridge, UK; 6 Unit of Internal Medicine, Department of Medicine, University Hospital of Lausanne, Lausanne, Switzerland; 7 Department of Medicine, Boston VA Healthcare System and Beth Israel Deaconess Medical Center, Harvard Medical School, Boston, USA; 8 United Kingdom Dementia Research Institute at Imperial College London, London, UK; 9 British Heart Foundation Centre for Research Excellence, Imperial College London, London, UK; 10 Unit of Cardiovascular and Nutritional Epidemiology, Institute of Environmental Medicine, Karolinska Institute, Stockholm, Sweden; 11 Unit of Medical Epidemiology, Department of Surgical Sciences, Uppsala University, Uppsala, Sweden; 12 Division of Systems Biology, Biomedical Research Foundation of the Academy of Athens, Athens, Greece

**Keywords:** Caffeine, Phenome-wide association study, Mendelian randomization, Osteoarthritis, Obesity

## Abstract

**Background:**

Caffeine is one of the most utilized drugs in the world, yet its clinical effects are not fully understood. Circulating caffeine levels are influenced by the interplay between consumption behaviour and metabolism. This study aimed to investigate the effects of circulating caffeine levels by considering genetically predicted variation in caffeine metabolism.

**Methods:**

Leveraging genetic variants related to caffeine metabolism that affect its circulating levels, we investigated the clinical effects of plasma caffeine in a phenome-wide association study (PheWAS). We validated novel findings using a two-sample Mendelian randomization framework and explored the potential mechanisms underlying these effects in proteome-wide and metabolome-wide Mendelian randomization.

**Results:**

Higher levels of genetically predicted circulating caffeine among caffeine consumers were associated with a lower risk of obesity (odds ratio (OR) per standard deviation increase in caffeine = 0.97, 95% confidence interval (CI) CI: 0.95—0.98, *p* = 2.47 × 10^−4^), osteoarthrosis (OR = 0.97, 95% CI: 0.96—0.98, P=1.10 × 10^−8^) and osteoarthritis (OR: 0.97, 95% CI: 0.96 to 0.98, *P* = 1.09 × 10^−6^). Approximately one third of the protective effect of plasma caffeine on osteoarthritis risk was estimated to be mediated through lower bodyweight. Proteomic and metabolomic perturbations indicated lower chronic inflammation, improved lipid profiles, and altered protein and glycogen metabolism as potential biological mechanisms underlying these effects.

**Conclusions:**

We report novel evidence suggesting that long-term increases in circulating caffeine may reduce bodyweight and the risk of osteoarthrosis and osteoarthritis. We confirm prior genetic evidence of a protective effect of plasma caffeine on risk of overweight and obesity. Further clinical study is warranted to understand the translational relevance of these findings before clinical practice or lifestyle interventions related to caffeine consumption are introduced.

**Supplementary Information:**

The online version contains supplementary material available at 10.1186/s12916-024-03298-y.

## Background

Caffeine (1,3,7-trimethylxanthine) is a plant-based bioactive compound present in a wide range of frequently consumed dietary sources such as coffee, soft drinks, tea, and chocolate [1]. The widespread availability of caffeine, and its effects on alertness, endurance, concentration, and productivity has made it one of the most utilized psychoactive stimulants in the world [1, 2].

Caffeine effectively permeates all cellular membranes, including the blood-brain barrier, and is able to exert broad and rapid effects across organ systems. These effects are primarily driven by the role of caffeine and two of its downstream metabolites, paraxanthine, and theophylline, as antagonists of adenosine receptors in the brain, adipose tissue, and the renal, cardiovascular, respiratory, and gastrointestinal systems [3].

Previous work has leveraged genetic variants affecting caffeine metabolism to identify evidence of causal effects of higher plasma caffeine levels on reducing adiposity and the risk of type 2 diabetes [4]. However, genetic and traditional epidemiological investigations into the effect of caffeine consumption have had contrasting findings [5, 6]. This discrepancy may be explained by the observation that individuals who metabolize caffeine at a slower rate have higher plasma levels of caffeine [4, 5, 7]. Thus, consideration of caffeine consumption as a proxy for the biological effects of plasma caffeine itself may give opposing results because individuals that have lower plasma caffeine levels (due to faster metabolism) tend to have higher caffeine intake [6].

Understanding the broader clinical effects of plasma caffeine using observational consumption data has been limited by the inability to disentangle the effect of caffeine itself from the co-occurring bioactive compounds in caffeinated foods and beverages [5]. Inferring causal effects from dietary data are further hindered by confounding through the various lifestyle and environmental factors that are associated with caffeine consumption [6]. Finally, interindividual differences in caffeine metabolism can also affect its levels of consumption, potentially biasing observational findings [3]. These differences are partially driven by genetic variants near the primary caffeine metabolizing protein gene, cytochrome P450 isoform 1A2 (*CYP1A2*), and its transcriptional regulator, aryl hydrocarbon receptor (*AHR*). Such genetic variants that affect caffeine metabolism can be used as instrumental variables in the Mendelian randomization (MR) paradigm for studying the effects of small variations in plasma caffeine levels over the course of a lifetime [5, 8].

Given the broad physiological processes impacted by plasma caffeine levels [9], we sought to systematically investigate its clinical effects by performing a phenome-wide association study (PheWAS), where we looked to identify all the clinical outcomes associated with genetic predictors of plasma caffeine levels. We further explored the mechanisms potentially underlying any identified associations by performing proteome-wide and metabolome-wide MR analyses.

## Methods

### Study overview

**Fig. 1:**
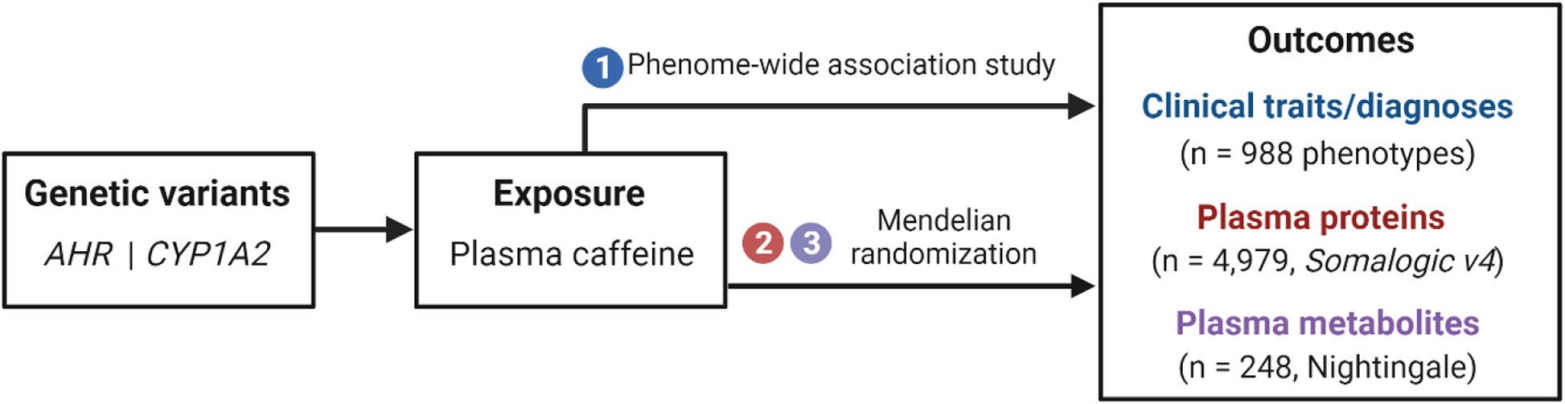
Study design. Abbreviations: AHR: aryl hydrocarbon receptor, CYP1A2: cytochrome P450 isoform 1A2. The schematic was created using Biorender.com

We generated a weighted genetic risk score (GRS) for plasma caffeine using two genetic variants independently associated with plasma caffeine levels. Using this GRS, we conducted a PheWAS using 988 clinical traits captured in the UK Biobank (UKB) cohort, to identify health outcomes that associate with plasma caffeine levels. For any novel PheWAS findings, we performed a two-sample MR replication analysis. Finally, we performed a proteome-wide and metabolome-wide MR analysis to explore the potential mechanisms underlying the effects of plasma caffeine on previously reported and novel traits. Figure 1 depicts the study design. Appropriate ethical approval and participant consent for all data used in this work was obtained in the original studies.

### Instrument selection for plasma caffeine

Genetic association data for plasma caffeine were retrieved from a genome-wide association study (GWAS) meta-analysis conducted by Cornelis et al., comprising a total of 9876 participants mostly of European descent, with ages ranging from 47 to 71 years old [7, 10]. Plasma caffeine measures were standardized by each study, and participants were required to fast prior to the collection of blood samples. Most studies reported association estimates adjusted for smoking status, age, sex, and genetic principal components. Details of the GWAS methods can be found in the supplemental material of the original publication. We instrumented plasma caffeine using the single-nucleotide polymorphisms (SNPs) within the *CYP1A2* (Chr15:75041185-75048543, GRCh37/hg19 by Ensembl) and *AHR* (Chr7:17338246-17385776, GRCh37/hg19 by Ensembl) that had the strongest associations with plasma caffeine levels. Specifically, we used rs2472297, the lead variant for plasma caffeine within the *CYP1A2* locus, and rs4410790, the corresponding lead *AHR*variant. These genes were chosen because of their relevance to caffeine metabolism [7]. These variants have been utilized as instrumental variables for plasma caffeine in previous MR studies [4, 11].

### Outcome data sources

#### Clinical traits

The UKB is a prospective cohort study initiated in 2006 that involved more than 500,000 participants between the ages of 40 to 69 years [12, 13]. The study obtained phenotypic data, genome-wide genotyping, and biological samples. Information on the genotyping process and data management has been reported elsewhere [14]. Individual level data in the UKB were used under UKB project application 236.

To conduct PheWAS in the UKB, International Classification of Diseases (ICD) versions 9 and 10 were used to identify cases in the Hospital Episode Statistics data, with diagnoses linked to the phenotype code (phecode) grouping system to allow for better identification of clinically relevant phenotypes [15]. The PheWAS was conducted using R package “PheWAS” v.0.99.5.5 [16].

#### Osteoarthritis

GWAS summary data for osteoarthrosis and osteoarthritis were obtained from a meta-analysis conducted by Boer et al. on 177,517 any-site osteoarthritis cases and 649,173 controls across 21 cohorts [17, 18]. Participants were defined as osteoarthritis cases based on self-reported information, clinical diagnosis, electronic ICD version 10 codes, or radiographic evidence, depending on data availability. Osteoarthrosis and osteoarthritis were used interchangeably in accordance with the ICD10 classification. Each cohort conducted their own GWAS analysis using cohort-specific covariates. Additional information about the GWAS meta-analysis can be found in the supplementary material of the original article.

#### Post-menopausal bleeding

GWAS summary data for post-menopausal bleeding were obtained from the FinnGen consortium data freeze round 9, consisting of 11,582 cases and 107,564 controls [19, 20]. Postmenopausal bleeding cases were ascertained using ICD-10 code N95.0.

#### Plasma proteins

Genetic association data for plasma protein levels were obtained from the Ferkingstad et al. GWAS that included 4907 aptamer-based protein measures in 35,559 Icelanders [21, 22]. Using plasma samples collected between 2000 and 2019, plasma samples were measured with the SomaScan version 4 assay (SomaLogic). The average participant age was 55 ± 17 years and 57% were women. The levels of the 4907 aptamers were adjusted for age, sex, and sample age, and then standardized using rank-inverse normal transformation. Genetic association estimates were obtained implementing a linear mixed model.

#### Plasma metabolites

Nuclear magnetic resonance spectroscopy was used by Nightingale Health to quantify 249 metabolic measures (168 in absolute levels and 81 metabolite ratios) from non-fasting plasma samples of approximately 120,000 randomly selected UKB participants. We extracted GWAS summary statistics for 115,078 participants of European ancestry using the OpenGWAS platform (identifier met-d-) [23, 24].

### Statistical analysis

#### Phenome-wide association study

A weighted GRS for plasma caffeine was created by adding up the number of plasma caffeine-increasing alleles for the two instrument variants used, with each allele multiplied by its corresponding beta estimate for the association with plasma caffeine levels from the Cornelis et al. GWAS [7, 10]. A logistic regression was performed for each phecode against the standardized plasma caffeine GRS and adjusting for age, sex, and the first 10 genetic principal components. Only phecodes with 200 or more cases were included in the analysis. Beta estimates were reported per one standard deviation (SD) increase in the standardized plasma caffeine GRS. Associations in the PheWAS were deemed statistically significant if they had a false discovery rate (FDR) *p*-value below 5%, accounting for multiple testing.

#### Mendelian randomization analysis

We leveraged the two-sample MR paradigm to explore the effect of plasma caffeine levels on osteoarthritis, postmenopausal bleeding, plasma proteins and plasma metabolites. The genetic associations with both the exposure (plasma caffeine) and outcomes were aligned by matching the effect alleles, utilizing the ‘TwoSampleMR’ v.0.6.0 R package [25]. The random-effects inverse-variance weighted method was used to estimate the MR effects [26]. To quantify heterogeneity in the MR estimates, we calculated the Cochran’s Q statistic [27]. We considered MR associations to be statistically significant if they survived a 5% FDR correction for the respective 4907 (proteome-wide), and 249 (metabolome-wide) tests. The MR effect estimates were reported per one SD increase in genetically predicted plasma caffeine levels. The biological roles of plasma caffeine-associated proteins were further investigated using the Uniprot (www.uniprot.org) [28] and STRING (https://string-db.org/) databases. Pathway and gene-enrichment analyses were performed using the SomaScan version 4 aptamer panel as reference.

#### Stratified analyses

If the instrumental variable is linked to the outcome through pathways independent of the exposure, bias may be introduced in the MR estimates. To investigate the presence of possible pleiotropic effects that might introduce bias, we stratified participants in UKB based on whether they reported consuming coffee or tea (Data fields 100240 and 100390). If the genetic variants are acting through plasma caffeine levels, we would expect their associations with disease risk to be attenuated in individuals that report not taking coffee or tea, as these individuals would have lower or absent plasma caffeine. The absence of such attenuation might suggest that the variants are acting through pathways independent of plasma caffeine levels. In each stratum, we explored the associations between a weighted GRS for circulating caffeine levels (constructed using the two caffeine metabolism instruments, as above), and osteoarthritis risk and body mass index (BMI). In logistic regression models, we used age, sex and the first 10 genetic principal components as covariates. The osteoarthritis binary variable was generated using the health episode statistics (Data field 41270) and extracting ICD-10 codes M15 to M19. BMI was measured on physical assessment (Data field 21001). For consistency with the PheWAS and MR, the analysis was restricted to European ancestry individuals in the UKB, resulting in 198,831 participants with complete data for these analyses.

#### Mediation analysis

Previous work has identified evidence supporting the effects of plasma caffeine on reducing BMI [4]. To investigate whether changes in BMI may be mediating any effect of plasma caffeine levels on the considered outcomes, we performed two-step network summary data MR mediation analysis. Specifically, the MR estimate for the effect of plasma caffeine on BMI was multiplied by the MR estimate for the effect of BMI on the outcome, to estimate the effect of plasma caffeine levels on the outcome that is mediated through BMI. This was divided by the MR estimate for the total effect of plasma caffeine on the outcome to derive the proportion of the effect that is mediated through BMI. MR analyses were performed as described above. Mediation analysis standard errors were calculated using the propagation of error method. GWAS summary data for BMI were obtained from a study of 806,834 European ancestry participants [29, 30]. Genetic instruments for BMI were selected as SNPs that associated with BMI at *p* < 5 × 10^−8^ and were weakly correlated (*r*^2^ < 0.01). Pruning was performed using the European 1000G reference panel within the ‘TwoSampleMR’ package of R.

#### Relationship between caffeine metabolism and caffeine consumption

To investigate the relationship between caffeine metabolism and caffeine consumption, we performed bi-directional random-effects inverse-variance weighted MR, as described above. Genetic association data and instruments for caffeine metabolism were taken from the same source described above [7, 10]. Caffeine consumption was proxied using self-reported coffee intake, for which genetic association data were taken from 428,860 individuals in UK Biobank, obtained through the OpenGWAS portal (identifier ukb-b-5237) [23, 24]. Instruments were selected as uncorrelated variants (*r*^2^ < 0.001) that associated with coffee intake at *p* < 5 × 10^−8^.

#### Genetically predicted plasma caffeine levels and comparative body size at age 10 years

Any association of the genetic variants used as instruments for plasma caffeine levels with comparative body size at 10 years, at which age individuals would be less likely consume much caffeine, would suggest that any observed association of these variants with BMI in adulthood is likely attributable to pleiotropic effects unrelated to plasma caffeine. This possibility was investigated using genetic association data for comparative body size at age 10 years in 454,718 individuals in UK Biobank, obtained through the OpenGWAS platform (identifier ukb-b-4650) [23, 24].

#### Colocalisation analysis

Colocalisation analysis was performed to test whether there was evidence of a shared causal variant underlying the MR association between plasma caffeine levels and BMI at the *CYP1A2* and *AHR*genes, respectively [4, 31]. This would help exclude genetic confounding through a variant in linkage disequilibrium as a potential explanation for the observed MR association. The default settings of the ‘coloc’ statistical software package version 5.2.3 were used [31].

## Results

The variance in plasma caffeine levels explained by the genetic instruments was 0.76% for rs2472297 in *CYP1A2* and 0.56% for rs4410790 in *AHR*.

**Fig. 2:**
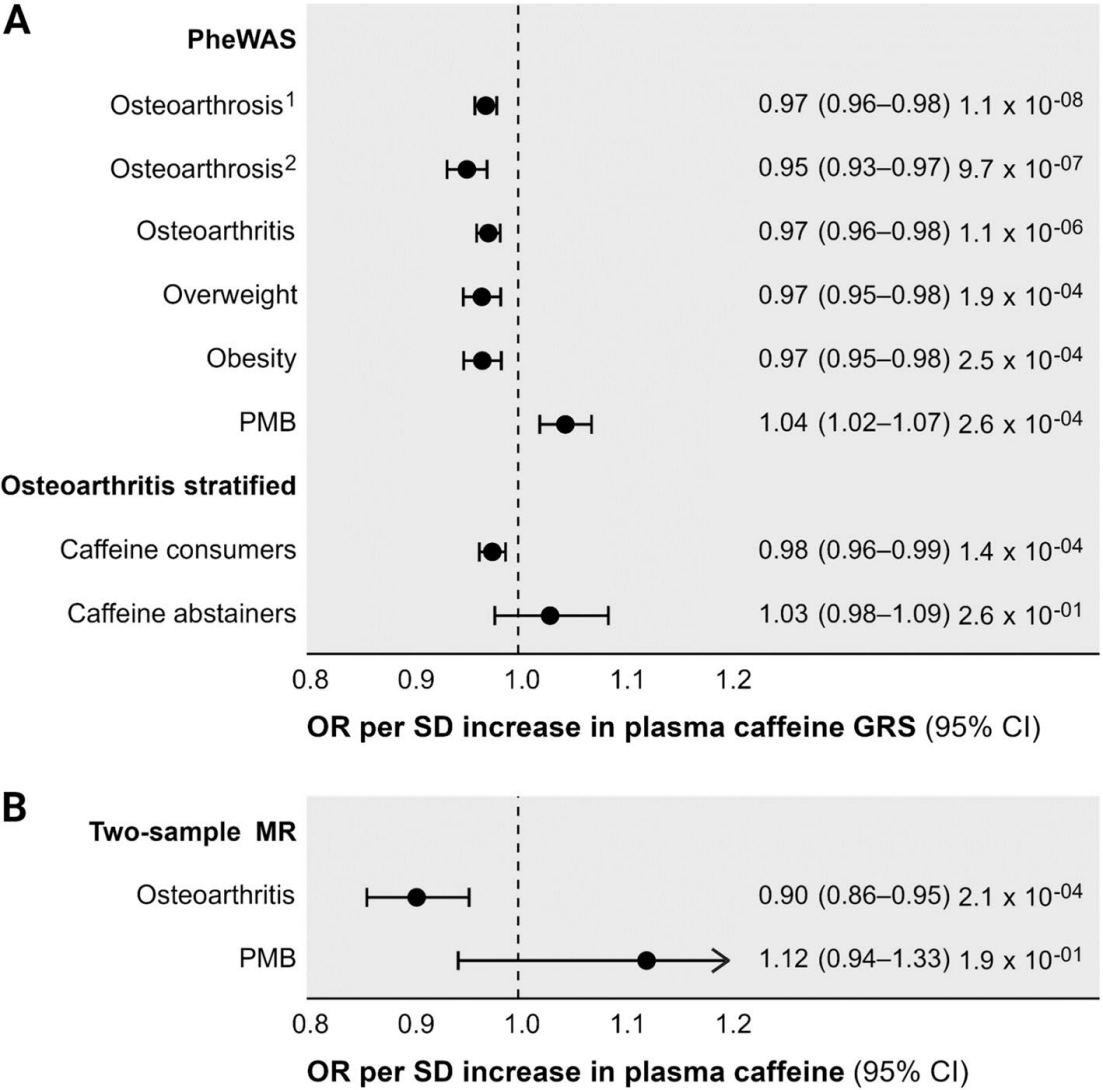
Disease odds ratio per standard deviation increase in the plasma caffeine genetic risk score (**A**) and genetically predicted plasma caffeine levels (**B**). The PheWAS and stratified analysis considered associations of a weighted genetic risk score for plasma caffeine, whereas the two-sample MR performed inverse-variance weighted MR analysis using genetic variants predicting plasma caffeine levels. Abbreviations: CI: confidence interval, GRS: genetic risk score, MR: Mendelian randomization, OA: osteoarthritis OR: odds ratio, PheWAS: Phenome-wide association analysis, PMB: postmenopausal bleeding, SD: standard deviation. Notes: ^1^Defined as International Classification of Diseases (ICD) version 10 codes M15–M19, ^2^Defined as ICD10 codes M19. Estimates are presented as the odds ratios (95% confidence interval) per standard deviation change in the genetic risk score of caffeine (**A**), and genetically predicted plasma caffeine levels (**B**). Estimate *p*-values are presented on the far right

In our hypothesis-free PheWAS, higher predicted circulating caffeine from the GRS was associated with a significantly lower risk of osteoarthrosis/osteoarthritis and overweight/obesity outcomes (Fig. 2, Additional file 1: Table S1). The weighted GRS for plasma caffeine was also associated with a higher risk of postmenopausal bleeding (Fig. 2).

In line with our PheWAS findings, two-sample MR analyses identified a 10% lower osteoarthritis (comprising osteoarthrosis or osteoarthrosis at any site) risk (odds ratio 0.90, 95% confidence interval [CI] 0.86 to 0.95, *p* = 2 × 10^−4^) per SD higher genetically predicted plasma caffeine. In MR mediation analyses, we estimated that 33.4% (95% CI 13.9% to 52.9%) of the effect of plasma caffeine on osteoarthritis risk was mediated through BMI. There was no two-sample MR evidence that plasma caffeine levels affected risk of postmenopausal bleeding in the FinnGen consortium (odds ratio 1.21, 95% CI 0.94 to 1.33, *p* = 0.19).

In analyses stratified by self-reported of coffee or tea consumption, there was statistically significant evidence of an association between genetically predicted plasma caffeine and osteoarthritis risk in caffeine consumers, but not in abstainers, although for the latter group CIs were wide reflecting the relatively small sample size (Fig. 2). For BMI, there was also a significant association with genetically predicted plasma caffeine levels in self-reported consumers of tea or coffee (− 0.05 SD change in BMI per SD increase in plasma caffeine, 95% CI − 0.07 to − 0.03, *p* = 2.5 × 10^−6^), but not in abstainers (− 0.09, 95% CI − 0.18 to 0.01, *p* = 0.08). Again, CIs were relatively wide for the latter group, reflecting the relatively small sample size.

Seven of the 4907 plasma proteomic markers investigated were associated with genetically predicted plasma caffeine levels). These included lower levels of endoplasmic reticulum oxidoreductase 1-beta (ERO1LB), hexosaminidase subunit beta (HEXB), immunoglobulin heavy constant gamma-4 (IGHG4), macrophage migration inhibitory factor (MIF), neuroepithelial cell transforming gene-1 (NET1) and protein phosphatase-1 regulatory subunit-3b (PPP1R3B), and higher levels of SLIT and NTRK like family member 3 (SLITRK3) with higher genetically predicted plasma caffeine. Table 1 provides the association estimates and broad function of each protein. There were no overrepresented biological pathways or ontologies among these proteins.

**Table 1:** Mendelian randomization associations between genetically predicted plasma caffeine levels and circulating proteins

**Protein**	**Estimate (95% CI)**	***p***_**fdr**_	Protein function*
Immunoglobulin heavy constant-γ4	− 0.49 (− 0.58; − 0.40)	6.1×10^−22^	**Adaptive immunity**. Triggers clonal expansion and differentiation of B-lymphocytes and eliminates bound antigens [25].
Neuroepithelial cell transforming-1	− 0.34 (− 0.43; − 0.25)	7.1×10^−10^	**GTP activity**. Acts as guanine nucleotide exchange factor for RhoA GTPase [26].
Endoplasmic reticulum oxidoreductase-1β	− 0.29 (− 0.38; − 0.20)	1.5×10^−06^	**Protein metabolism**. Regulates protein folding in the endoplasmic reticulum, including that of proinsulin [27].
Macrophage migration inhibitory factor	− 0.26 (− 0.35; − 0.17)	2.0×10^−05^	**Innate immunity**. Pro-inflammatory cytokine that regulates macrophage function [28].
SLIT and NTRK-like family member-3	0.26 (0.16; 0.35)	5.6×10^−05^	**Synaptic regulation**. Suppresses neurite outgrowth [29].
Protein phosphatase-1 regulatory subunit 3β	− 0.24 (− 0.33; − 0.15)	3.8×10^−04^	**Glycogen metabolism**. Limits the breakdown of glycogen and increases its synthesis under stress [30].
Hexosaminidase subunit-β	− 0.20 (− 0.29; − 0.11)	9.2×10^−03^	**Glycogen metabolism.**Responsible for the hydrolysis of neutrally charged oligosaccharides [31].

Betas represent the standard deviation change in relative protein abundance per standard deviation increase in plasma caffeine level. *Function as per the Uniprot database (www.uniprot.org/ ) [22] and published literature

Evidence of the broader effect of plasma caffeine was observed in our metabolomic analyses, where 91 of the 249 tested circulating metabolites were significantly associated with instrumented plasma caffeine levels (Additional file 1: Table S2). In these analyses, instrumented plasma caffeine negatively associated with the total lipid content and particle concentration of very small to medium-sized very low-density lipoproteins (VLDLs), low-density lipoproteins (LDLs, all sizes) and their primary carrier apolipoprotein-B (ApoB), intermediate-density lipoproteins (IDLs) and small high-density lipoproteins (HDLs). We also observed a reduction in the inflammatory marker glycoprotein acetyls (glycA), lower bioactive lipids phosphatidylcholines and phosphoglycerides, and ketone bodies acetoacetate and 3-hydroxybutyrate, with higher levels of instrumented plasma caffeine.

The bi-directional MR investigating the relationship between plasma caffeine and coffee consumption identified evidence of an association between higher genetically predicted plasma caffeine and lower coffee consumption (*p* = 1.9 × 10^−45^), but not between genetically predicted coffee consumption and plasma caffeine levels (*p* = 0.17).

There was no strong evidence that genetically predicted plasma caffeine levels associate with comparative body size at age 10 years (*p* = 0.31), supporting that the observed MR association with adult BMI is unlikely to be attributable to pleiotropic effects unrelated to plasma caffeine.

There was strong genetic evidence of a shared causal variant for the association between plasma caffeine levels and BMI at the *CYP1A2* (posterior probability 98%) and *AHR* (posterior probability 99%) genes. This is consistent with the identified MR association being attributable to a true causal effect of plasma caffeine levels on BMI, rather than genetic confounding through a variant in linkage disequilibrium.

## Discussion

In this large PheWAS and MR study, we report an association between higher genetically predicted plasma caffeine levels and a lower risk of osteoarthritis. We also replicated prior findings suggesting the protective effect of higher plasma caffeine levels on obesity risk [6], with MR mediation analyses indicating that around a third of the protective effect of plasma caffeine on osteoarthritis risk was mediated through lower bodyweight. Although the PheWAS identified a potential association between plasma caffeine levels and risk of postmenopausal bleeding, this was not apparent in two-sample MR using data from an independent cohort. Finally, we report plasma caffeine-mediated changes in the plasma proteome and metabolome that further contextualize our primary findings.

Osteoarthritis is a widely prevalent, age-associated disease that involves progressive destruction of the synovial joint, including degeneration of the cartilage, synovial inflammation, and subchondral bone remodelling [32]. Factors contributing to osteoarthritis risk and severity overlap substantially with those of metabolic syndrome (obesity, hyperlipidemia, hypertension) [33]. While there are currently no disease-modifying treatments available for osteoarthritis, prevention and symptom management strategies center on lifestyle modifications including minimizing occupational strain, and weight maintenance [32].

**Fig. 3:**
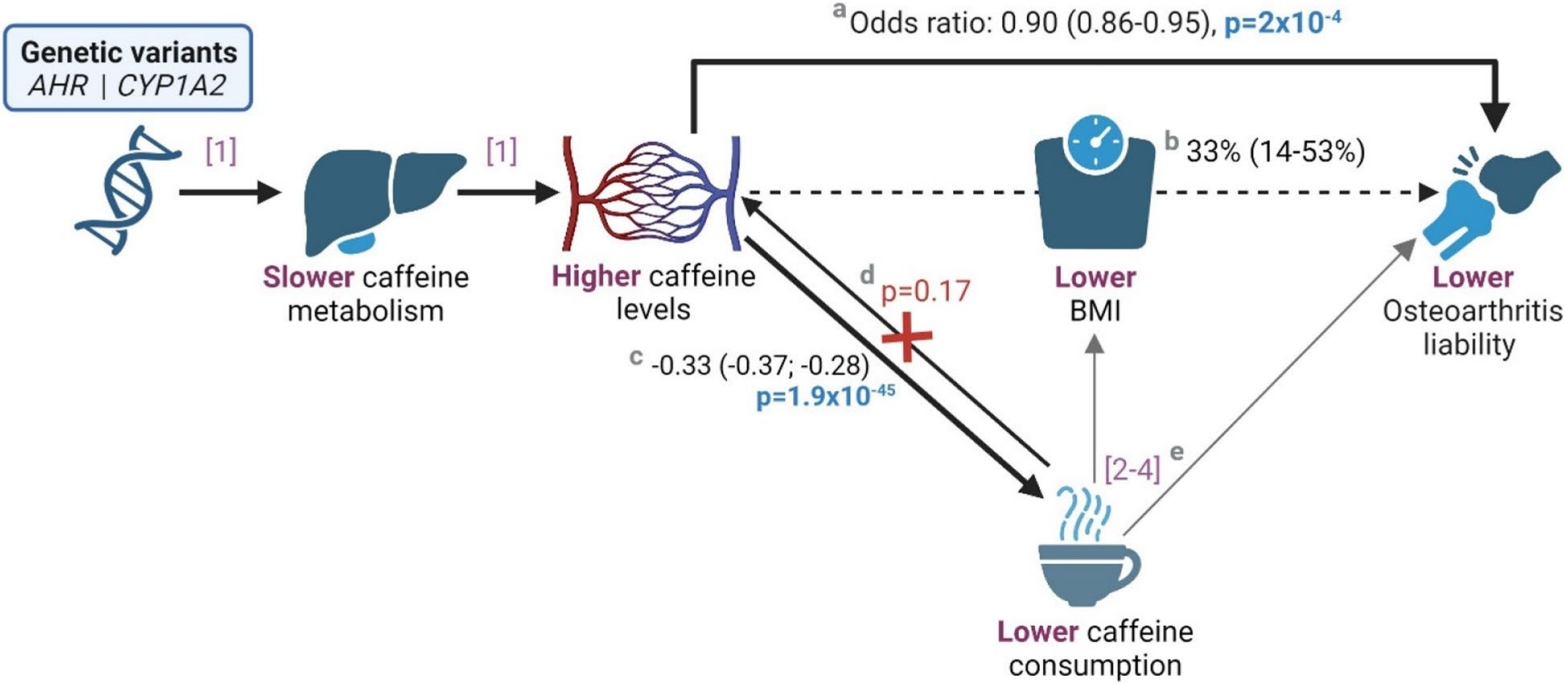
An illustrative mechanistic diagram showing the protective effect of genetically predicted plasma caffeine levels on osteoarthritis liability^(a)^, mediated through body mass index^(b)^. The figure extends to a representation of our findings that genetically predicted caffeine levels drive consumption behaviour^(c)^, but not vice versa^(d)^. In keeping with our findings, prior studies have reported a positive association between caffeine consumption and body mass index and osteoarthritis risk^(e)^. Mendelian randomization estimates are scaled per one standard deviation increase in plasma caffeine levels and are shown with their corresponding 95% confidence intervals in parenthesis. Citations included [1] Cornelis et al. (2016); [2] Nicolopoulos et al. (2020); [3] Lee (2018); [4] Zhang et al. (2021). Abbreviations: AHR: aryl hydrocarbon receptor, BMI: body mass index, CYP1A2: cytochrome P450 isoform 1A2. This figure was created using Biorender.com

The observational literature on the role of caffeine in osteoarthritis remains inconclusive but largely reports caffeine intake as a risk factor of osteoarthritis, particularly when consumed during key growth phases, including maternally in pregnancy [34–36]. Two hypothesis-driven MR studies have previously reported a positive association between genetically instrumented coffee consumption and osteoarthritis risk [37, 38]. Consistent observations were subsequently reported in a hypothesis-free MR and PheWAS analysis of caffeine consumption [39]. The latter investigation also reported that instrumented coffee consumption was associated with an increase in the risk of obesity and a reduction in postmenopausal bleeding risk. Although these results may seem contradictory to our findings of a caffeine-mediated reduction in osteoarthritis and obesity risk and an increase in the risk of postmenopausal bleeding, nuances in the exposure definitions are crucial to understanding the discrepancy. Our investigation instruments plasma caffeine, and more specifically, the genetically predicted ability to metabolize caffeine. Prior work has shown higher plasma levels of caffeine in those who metabolize caffeine at a slower rate, to the extent that consumption and plasma levels are opposing [4, 5, 7]. Our current analysis similarly supports that higher plasma caffeine may have a causal effect on reducing caffeine consumption. Thus, consideration of caffeine consumption as a proxy for the biological effects of caffeine itself may give opposing results because individuals that have lower plasma caffeine levels (due to faster metabolism) tend to have higher caffeine intake [6]. A summary of the main findings of our current study in the context of previous research is presented in Fig. 3.

Our proteome-wide MR triangulated evidence for previously reported mechanisms of caffeine action in an investigation of human hepatocytes. Specifically, our evidence of a caffeine-mediated reduction in plasma ERO1LB, HEXB, and PPP1R3B complemented observed effects on hepatocellular glycogen and protein metabolism [40]. Caffeine-mediated upregulation of guanosine triphosphate hydrolase enzyme (GTPase) in human hepatocytes also aligns with the changes observed in plasma NET1 and SLITRK3 [40], both of which are involved in guanyl-nucleotide exchange activity [28, 41].

A substantial body of experimental and observational evidence supports the effect of lowering MIF levels in reducing osteoarthrosis severity through limiting tissue damage related to its pro-inflammatory effects [42, 43]. Our finding of lower IGHG4 further supports a caffeine-related reduction in inflammatory signalling [28]. Likewise, our metabolome-wide MR analysis reflected a caffeine-related reduction in pro-inflammatory lipids. Specifically, we observed a negative association between instrumented plasma caffeine levels and VLDL, IDL, LDL, ApoB, saturated fatty acids, and phosphoglycerides, all of which contribute to chronic low-grade inflammation [44]. A concurrent reduction in the inflammatory marker GlycA was also observed, and lower levels of phosphatidylcholine, a bioactive lipid related to platelet activation, symptom severity in patients with osteoarthrosis [45]. Caffeine-associated changes in amino acid levels may be indicative of less tissue damage (e.g. lower circulating branch-chain amino acids) [46] and lower synovial need for reabsorption and repair resources (e.g. higher circulating glycine, which is a major component of cartilage) [47].

Regarding our UKB PheWAS finding of an increased risk of postmenopausal bleeding related to higher genetically predicted plasma caffeine levels, this did not replicate when performing MR using GWAS summary data from the independent FinnGen consortium. Given that more postmenopausal bleeding cases were available in the FinnGen data, this is unlikely to be attributable to statistical power. Potential explanations include misspecification of diagnoses in one or both datasets, or that the PheWAS finding in UKB was a false positive.

The MR framework enabled us to provide rapid and cost-effective clinically relevant insight into the effects of plasma caffeine on a broad range of traits and diagnoses. Using genetic instruments of the exposure (plasma caffeine) instead of the exposure itself strengthens the robustness of our findings by circumventing common limitations of epidemiological study designs, such as reverse causality and confounding. It also allowed for follow-up and individual-level data sensitivity analyses of our primary finding, namely a potential effect of higher plasma caffeine on reducing osteoarthritis risk. At the same time, our reliance on genetic evidence limits the clinical translatability of our findings in that they reflect small lifelong effects that do not take the pharmacokinetic properties of caffeine consumption in everyday life into account. Our findings should not be extrapolated to predict the size of acute clinical or public health benefits of specific interventions. Further, the MR paradigm requires that the variants selected as instruments affect the outcome only through the exposure, and not some pleiotropic pathway. Although we attempted to test this in our individual participant data analysis that stratified the UKB population based on whether or not they reported drinking tea or coffee, estimates for osteoarthritis risk overlapped in consumers and non-consumers and further the findings may be vulnerable to collider bias, preventing us from drawing strong conclusions on the validity of the findings based on this analysis. In contrast, we did not identify any association of the instruments for plasma caffeine levels with comparative body size at age 10 years, supporting that the observed association with adult BMI is unlikely to be attributable to pleiotropic effects. The colocalization analysis additionally supported that the observed MR association between plasma caffeine and BMI was attributable to shared causal variants at the *CYP1A2* and *AHR* gene loci respectively, and not genetic confounding through variants in linkage disequilibrium. While incorporating large, open access datasets provided us with a wealth of insight to inform further clinical study, these data are inherently limited by their lack of ethnic and cultural diversity and largely represent individuals of European ancestry residing in Western societies. The benefit of larger sample sizes also comes at a cost of reduced phenotypic granularity and lack of specificity in the case of diagnostically complex conditions like osteoarthritis and osteoarthrosis.

## Conclusions

In conclusion, we use the wealth of genetic association data related to clinical, proteomic and metabolomic traits to provide complementary evidence supporting the potential causal effects of plasma caffeine concentrations on a broad range of health outcomes. We find evidence supporting the protective effects of plasma caffeine on osteoarthritis risk, and further support existing genetic evidence linking higher plasma caffeine with lower bodyweight. Given the widespread consumption of caffeine-containing beverages and the global burden of obesity and osteoarthritis, our findings are of considerable public health interest and should be used to prioritize further research efforts in this area. Further clinical study in the form of randomized trials may now be warranted to understand the translational relevance of these findings, before any potential clinical practice or lifestyle interventions related to caffeine consumption are introduced.

### Supplementary Information

**Additional file 1:** **Table S1.** Phenome-wide association estimates per standard deviation unit increase in standardized plasma caffeine level genetic risk score. **Table S2.** Mendelian randomization estimates for the association of one standard deviation unit increase in genetically predicted plasma caffeine with plasma metabolite levels and ratios.

## Data Availability

UK Biobank individual level data used in this work can be accessed after applying for access at https://www.ukbiobank.ac.uk/enable-your-research/apply-foraccess. All genetic association data used in this work were obtained from publicly available sources. Link to datasets: Plasma caffeine: https://pubmed.ncbi.nlm.nih.gov/27702941/. Osteoarthritis: https://msk.hugeamp.org/downloads.html. FinnGen: https://www.finngen.fi/en/access_results. DeCODE plasma proteins: https://www.decode.com/summarydata/. NMR metabolic profiles: https://gwas.mrcieu.ac.uk/. Body mass index: https://zenodo.org/records/1251813#.XCLJ7vZKhE4.

## Abbreviations

ApoB: Apolipoprotein-B
CI: Confidence interval
glycA: Glycoprotein acetyls
GWAS: Genome-wide association study
HDL: High-density lipoproteins
ICD: International Classification of Diseases
IDL: Intermediate-density lipoproteins
LDL: Low-density lipoprotein
MR: Mendelian randomization
PheWAS: Phenome-wide association study
SD: Standard deviation
SNP: Single-nucleotide polymorphism
UKB: UK Biobank
VLDL: Very low-density lipoproteins
