# Supplementary material for: Genetic investigation into the broad health implications of caffeine: evidence from phenome-wide, proteome-wide and metabolome-wide Mendelian randomization
Source: BMC Med. 2024 Feb 20;22:81. doi: 10.1186/s12916-024-03298-y (PMC10880284; doi:10.1186/s12916-024-03298-y)
Supplement: Supplementary file 1 — Additional file 1: Table S1. Phenome-wide association estimates per standard deviation unit increase in standardized plasma caffeine level genetic risk score. Table S2. Mendelian randomization estimates for the association of one standard deviation unit increase in genetically predicted plasma caffeine with plasma metabolite levels and ratios. [file 12916_2024_3298_MOESM1_ESM.docx]

**Table S1**. Phenome-wide association estimates per standard deviation unit increase in standardized plasma caffeine level genetic risk score.

| **Trait description** | **OR (95% CI)** | **p-value** | **N cases** |
| --- | --- | --- | --- |
| **Circulatory system** | | | |
| Abdominal aortic aneurysm | 1.02 (0.96; 1.08) | 0.55 | 1,116 |
| Abnormal function study of cardiovascular system | 0.95 (0.87; 1.04) | 0.27 | 541 |
| Abnormal heart sounds | 1.00 (0.95; 1.06) | 0.91 | 1,243 |
| Acute vascular insufficiency of intestine | 0.99 (0.88; 1.11) | 0.87 | 306 |
| Aneurysm of other specified artery | 0.97 (0.85; 1.11) | 0.66 | 235 |
| Angina pectoris | 0.98 (0.97; 1.00) | 0.02 | 16,400 |
| Anomalous atrioventricular excitation | 1.10 (0.96; 1.26) | 0.17 | 222 |
| Aortic aneurysm | 0.99 (0.94; 1.04) | 0.69 | 1,674 |
| Arrhythmia (cardiac) NOS | 0.99 (0.92; 1.06) | 0.75 | 883 |
| Arterial embolism and thrombosis | 0.95 (0.89; 1.02) | 0.13 | 868 |
| Arterial embolism and thrombosis of lower extremity artery | 0.97 (0.90; 1.06) | 0.54 | 558 |
| Arteritis NOS | 1.03 (0.92; 1.16) | 0.56 | 318 |
| Atherosclerosis | 0.99 (0.93; 1.05) | 0.63 | 1,161 |
| Atherosclerosis of the extremities | 0.97 (0.90; 1.03) | 0.29 | 907 |
| Atrial fibrillation | 1.03 (0.97; 1.08) | 0.34 | 1,469 |
| Atrial fibrillation and flutter | 0.99 (0.97; 1.01) | 0.23 | 16,861 |
| Atrial flutter | 0.96 (0.85; 1.09) | 0.53 | 272 |
| Atrioventricular [AV] block | 0.98 (0.94; 1.02) | 0.28 | 2,573 |
| Atrioventricular block, complete | 1.00 (0.93; 1.08) | 0.91 | 756 |
| Bundle branch block | 1.00 (0.96; 1.05) | 0.89 | 2,107 |
| Cardiac arrest | 1.04 (0.94; 1.15) | 0.42 | 428 |
| Cardiac arrest and ventricular fibrillation | 0.98 (0.91; 1.06) | 0.62 | 757 |
| Cardiac conduction disorders | 1.00 (0.98; 1.03) | 0.78 | 6,353 |
| Cardiac dysrhythmias | 0.99 (0.98; 1.00) | 0.20 | 26,450 |
| Cardiac pacemaker in situ | 0.99 (0.95; 1.04) | 0.79 | 2,427 |
| Cardiac pacemaker/device in situ | 0.99 (0.95; 1.03) | 0.69 | 2,628 |
| Cardiomegaly | 0.99 (0.95; 1.03) | 0.54 | 2,820 |
| Cardiomyopathy | 1.06 (1.00; 1.11) | 0.04 | 1,494 |
| Carditis | 0.98 (0.94; 1.03) | 0.49 | 2,102 |
| Cerebral aneurysm | 0.96 (0.87; 1.06) | 0.40 | 409 |
| Cerebral artery occlusion, with cerebral infarction | 1.01 (0.97; 1.05) | 0.68 | 2,965 |
| Cerebral atherosclerosis | 0.95 (0.84; 1.08) | 0.46 | 250 |
| Cerebral ischemia | 0.98 (0.95; 1.02) | 0.38 | 3,008 |
| Cerebrovascular disease | 0.99 (0.96; 1.01) | 0.26 | 7,706 |
| Chronic pulmonary heart disease | 1.00 (0.94; 1.06) | 0.93 | 1,017 |
| Chronic venous insufficiency [CVI] | 0.95 (0.83; 1.08) | 0.43 | 232 |
| Circulatory disease NEC | 1.00 (0.98; 1.01) | 0.86 | 16,157 |
| Congestive heart failure (CHF) NOS | 0.98 (0.94; 1.03) | 0.43 | 1,940 |
| Congestive heart failure; nonhypertensive | 1.00 (0.97; 1.03) | 0.93 | 5,177 |
| Coronary atherosclerosis | 0.99 (0.98; 1.00) | 0.15 | 22,539 |
| Disease of tricuspid valve | 1.04 (0.93; 1.15) | 0.51 | 352 |
| Endocarditis | 0.95 (0.88; 1.03) | 0.21 | 636 |
| Essential hypertension | 1.00 (0.99; 1.00) | 0.34 | 72,166 |
| First degree AV block | 0.96 (0.91; 1.02) | 0.23 | 1,164 |
| Giant cell arteritis | 0.94 (0.86; 1.03) | 0.17 | 471 |
| Heart failure NOS | 1.02 (0.98; 1.05) | 0.30 | 3,851 |
| Heart valve disorders | 1.01 (0.98; 1.03) | 0.63 | 6,318 |
| Heart valve replaced | 1.01 (0.96; 1.07) | 0.62 | 1,598 |
| Hemorrhoids | 1.01 (0.99; 1.04) | 0.37 | 6,232 |
| Hypertension | 1.00 (0.99; 1.00) | 0.36 | 72,436 |
| Hypertensive chronic kidney disease | 1.00 (0.94; 1.05) | 0.94 | 1,379 |
| Hypertensive heart and/or renal disease | 0.99 (0.94; 1.05) | 0.84 | 1,552 |
| Hypotension | 1.00 (0.98; 1.03) | 0.72 | 6,585 |
| Hypotension NOS | 1.00 (0.97; 1.03) | 0.96 | 3,870 |
| Iatrogenic hypotension | 1.00 (0.87; 1.14) | 0.96 | 229 |
| Ill-defined descriptions/and complications of heart disease | 0.95 (0.87; 1.04) | 0.27 | 548 |
| Intracerebral hemorrhage | 1.00 (0.93; 1.08) | 0.94 | 786 |
| Intracranial hemorrhage | 0.98 (0.93; 1.03) | 0.39 | 1,549 |
| Ischemic Heart Disease | 0.99 (0.98; 1.01) | 0.26 | 33,878 |
| Left bundle branch block | 1.02 (0.97; 1.07) | 0.46 | 1,869 |
| Mitral valve disease | 0.97 (0.87; 1.08) | 0.57 | 344 |
| Mitral valve stenosis and aortic valve stenosis | 1.04 (0.97; 1.13) | 0.29 | 695 |
| Myocardial infarction | 0.99 (0.97; 1.01) | 0.25 | 9,142 |
| Noninfectious disorders of lymphatic channels | 0.95 (0.89; 1.02) | 0.19 | 823 |
| Nonrheumatic aortic valve disorders | 0.99 (0.96; 1.03) | 0.57 | 3,211 |
| Nonrheumatic mitral valve disorders | 1.02 (0.98; 1.06) | 0.41 | 2,685 |
| Nonspecific chest pain | 1.00 (0.90; 1.11) | 0.99 | 359 |
| Occlusion and stenosis of precerebral arteries | 0.98 (0.92; 1.03) | 0.38 | 1,293 |
| Occlusion of cerebral arteries | 1.01 (0.97; 1.05) | 0.61 | 3,010 |
| Orthostatic hypotension | 1.00 (0.95; 1.05) | 0.95 | 1,825 |
| Other acute and subacute forms of ischemic heart disease | 1.00 (0.95; 1.05) | 0.99 | 1,442 |
| Other aneurysm | 0.99 (0.95; 1.04) | 0.78 | 2,151 |
| Other chronic ischemic heart disease, unspecified | 0.99 (0.97; 1.00) | 0.10 | 14,612 |
| Other disorders of arteries and arterioles | 0.99 (0.93; 1.04) | 0.63 | 1,307 |
| Other disorders of circulatory system | 1.00 (0.98; 1.02) | 0.90 | 16,324 |
| Other forms of chronic heart disease | 1.03 (0.99; 1.08) | 0.15 | 2,202 |
| Other hypertrophic cardiomyopathy | 1.09 (0.94; 1.25) | 0.24 | 216 |
| Other specified cardiac dysrhythmias | 1.01 (0.98; 1.05) | 0.45 | 3,166 |
| Other venous embolism and thrombosis | 0.94 (0.83; 1.07) | 0.37 | 232 |
| Palpitations | 0.99 (0.95; 1.02) | 0.42 | 3,770 |
| Paroxysmal supraventricular tachycardia | 1.01 (0.97; 1.05) | 0.64 | 2,473 |
| Paroxysmal tachycardia, unspecified | 0.99 (0.96; 1.03) | 0.75 | 3,428 |
| Paroxysmal ventricular tachycardia | 0.96 (0.90; 1.02) | 0.16 | 1,045 |
| Pericarditis | 1.00 (0.95; 1.06) | 0.94 | 1,410 |
| Peripheral vascular disease | 0.98 (0.95; 1.01) | 0.26 | 3,903 |
| Peripheral vascular disease, unspecified | 0.98 (0.94; 1.01) | 0.21 | 2,751 |
| Phlebitis and thrombophlebitis | 0.93 (0.87; 0.98) | 0.01 | 1,136 |
| Phlebitis and thrombophlebitis of lower extremities | 0.93 (0.87; 1.00) | 0.04 | 815 |
| Polyarteritis nodosa and allied conditions | 0.98 (0.92; 1.05) | 0.63 | 997 |
| Precordial pain | 0.99 (0.96; 1.02) | 0.60 | 3,677 |
| Premature beats | 1.03 (0.95; 1.12) | 0.51 | 590 |
| Primary pulmonary hypertension | 1.06 (0.96; 1.17) | 0.24 | 431 |
| Primary/intrinsic cardiomyopathies | 1.06 (1.00; 1.11) | 0.04 | 1,457 |
| Pulmonary heart disease | 0.99 (0.93; 1.05) | 0.79 | 1,079 |
| Raynaud's syndrome | 1.00 (0.94; 1.06) | 0.95 | 1,169 |
| Rheumatic disease of the heart valves | 1.02 (0.98; 1.06) | 0.33 | 2,642 |
| Second degree AV block | 1.02 (0.94; 1.11) | 0.68 | 572 |
| Sinoatrial node dysfunction (Bradycardia) | 0.95 (0.87; 1.04) | 0.28 | 502 |
| Stricture of artery | 0.97 (0.91; 1.04) | 0.40 | 831 |
| Subarachnoid hemorrhage | 0.95 (0.88; 1.02) | 0.13 | 765 |
| Tachycardia NOS | 0.97 (0.93; 1.01) | 0.18 | 2,278 |
| Transient cerebral ischemia | 0.99 (0.94; 1.03) | 0.51 | 200 |
| Unstable angina (intermediate coronary syndrome) | 0.99 (0.97; 1.02) | 0.70 | 4,892 |
| Varicose veins | 1.02 (1.00; 1.04) | 0.05 | 10,296 |
| Varicose veins of lower extremity | 1.03 (1.00; 1.05) | 0.02 | 9,850 |
| Varicose veins of lower extremity, symptomtic | 1.00 (0.92; 1.07) | 0.91 | 734 |
| Vascular insufficiency of intestine | 1.02 (0.94; 1.10) | 0.62 | 696 |
| Ventricular fibrillation and flutter | 0.92 (0.82; 1.02) | 0.11 | 355 |
| Wegener's granulomatosis | 1.02 (0.89; 1.18) | 0.73 | 211 |
| **Congenital anomalies** | | | |
| Cardiac and circulatory congenital anomalies | 1.06 (1.00; 1.12) | 0.06 | 1,201 |
| Cardiac congenital anomalies | 1.07 (1.01; 1.14) | 0.03 | 1,027 |
| Cardiac shunt/ heart septal defect | 1.07 (0.98; 1.16) | 0.15 | 537 |
| Congenital anomalies of face and neck | 1.05 (0.93; 1.19) | 0.41 | 259 |
| Congenital anomalies of female genital organs | 1.05 (0.94; 1.17) | 0.41 | 341 |
| Congenital anomalies of genital organs | 1.03 (0.94; 1.12) | 0.58 | 490 |
| Congenital anomalies of limbs | 1.00 (0.90; 1.10) | 0.95 | 406 |
| Congenital anomalies of mouth/tongue | 0.99 (0.86; 1.13) | 0.85 | 212 |
| Congenital anomalies of muscle, tendon, fascia, and connective tissue | 1.01 (0.90; 1.14) | 0.83 | 301 |
| Congenital anomalies of urinary system | 0.99 (0.92; 1.06) | 0.76 | 772 |
| Congenital deformities of feet | 1.03 (0.89; 1.18) | 0.72 | 201 |
| Congenital musculoskeletal deformities of spine | 1.00 (0.88; 1.14) | 0.99 | 233 |
| Congenital osteodystrophies | 1.04 (0.94; 1.15) | 0.47 | 367 |
| Cystic kidney disease | 0.98 (0.89; 1.08) | 0.72 | 409 |
| Digestive congenital anomalies | 1.03 (0.95; 1.11) | 0.48 | 673 |
| Genitourinary congenital anomalies | 1.01 (0.96; 1.07) | 0.69 | 1,280 |
| Lower gastrointestinal congenital anomalies | 1.05 (0.94; 1.17) | 0.39 | 322 |
| Nervous system congenital anomalies | 1.02 (0.93; 1.12) | 0.64 | 494 |
| Neural tube defects | 1.01 (0.92; 1.11) | 0.84 | 434 |
| Other congenital musculoskeletal anomalies | 1.01 (0.93; 1.11) | 0.77 | 523 |
| Other specified congenital anomalies of kidney | 1.01 (0.90; 1.12) | 0.91 | 337 |
| Upper gastrointestinal congenital anomalies | 1.01 (0.91; 1.13) | 0.83 | 349 |
| Valvular heart disease/ heart chambers | 1.20 (1.06; 1.36) | 0.00 | 263 |
| **Dermatologic** | | | |
| Abnormal granulation tissue | 1.12 (1.01; 1.23) | 0.03 | 431 |
| Actinic keratosis | 1.01 (0.97; 1.06) | 0.51 | 2,237 |
| Atopic/contact dermatitis due to other or unspecified | 0.99 (0.95; 1.03) | 0.68 | 2,154 |
| Cellulitis and abscess of fingers/toes | 1.04 (0.96; 1.14) | 0.33 | 532 |
| Chronic dermatitis due to solar radiation | 0.96 (0.85; 1.09) | 0.51 | 251 |
| Chronic ulcer of leg or foot | 0.99 (0.94; 1.05) | 0.75 | 1,376 |
| Chronic ulcer of skin | 0.97 (0.93; 1.01) | 0.17 | 2,446 |
| Circumscribed scleroderma | 1.00 (0.91; 1.10) | 0.93 | 458 |
| Corns and callosities | 1.03 (0.91; 1.17) | 0.60 | 252 |
| Decubitus ulcer | 0.96 (0.90; 1.02) | 0.19 | 1,118 |
| Degenerative skin conditions and other dermatoses | 1.02 (0.99; 1.05) | 0.18 | 4,755 |
| Dermatitis due to solar radiation | 1.09 (0.98; 1.21) | 0.10 | 365 |
| Diffuse diseases of connective tissue | 0.98 (0.92; 1.04) | 0.46 | 979 |
| Diseases of hair and hair follicles | 0.96 (0.86; 1.07) | 0.43 | 354 |
| Diseases of nail, NOS | 1.03 (0.93; 1.15) | 0.56 | 336 |
| Diseases of sebaceous glands | 1.00 (0.98; 1.03) | 0.85 | 7,390 |
| Disorder of skin and subcutaneous tissue NOS | 0.97 (0.95; 1.00) | 0.06 | 5,391 |
| Disturbance of skin sensation | 0.99 (0.95; 1.03) | 0.67 | 2,656 |
| Dyschromia and Vitiligo | 1.07 (1.00; 1.14) | 0.04 | 938 |
| Erythematosquamous dermatosis | 1.02 (0.98; 1.06) | 0.29 | 2,794 |
| Erythematous conditions | 1.00 (0.96; 1.04) | 0.90 | 2,416 |
| Ingrowing nail | 0.95 (0.89; 1.02) | 0.18 | 796 |
| Keloid scar | 0.98 (0.85; 1.12) | 0.74 | 232 |
| Lupus (localized and systemic) | 0.98 (0.89; 1.08) | 0.69 | 477 |
| Other dyschromia | 1.09 (1.01; 1.18) | 0.02 | 731 |
| Other hypertrophic and atrophic conditions of skin | 1.00 (0.97; 1.03) | 0.80 | 4,602 |
| Other local infections of skin and subcutaneous tissue | 1.00 (0.95; 1.06) | 0.98 | 1,314 |
| Other specified erythematous conditions | 0.90 (0.80; 1.03) | 0.12 | 248 |
| Prurigo and Lichen | 1.08 (1.00; 1.16) | 0.05 | 714 |
| Pruritus and related conditions | 0.96 (0.89; 1.04) | 0.33 | 730 |
| Psoriasis | 1.03 (0.99; 1.07) | 0.21 | 2,275 |
| Psoriasis and related disorders | 1.03 (0.98; 1.07) | 0.23 | 2,328 |
| Psoriasis vulgaris | 1.03 (0.98; 1.08) | 0.23 | 1,689 |
| Psoriatic arthropathy | 1.00 (0.93; 1.08) | 0.91 | 738 |
| Pyogenic granuloma | 0.93 (0.83; 1.04) | 0.20 | 296 |
| Rash and other nonspecific skin eruption | 1.01 (0.97; 1.06) | 0.60 | 1,739 |
| Rosacea | 1.06 (0.94; 1.19) | 0.35 | 301 |
| Sarcoidosis | 1.02 (0.93; 1.11) | 0.72 | 588 |
| Scar conditions and fibrosis of skin | 0.97 (0.93; 1.01) | 0.19 | 2,040 |
| Sebaceous cyst | 1.01 (0.98; 1.03) | 0.67 | 7,320 |
| Seborrheic dermatitis | 1.02 (0.98; 1.06) | 0.31 | 2,772 |
| Seborrheic keratosis | 1.02 (0.98; 1.06) | 0.28 | 2,717 |
| Sicca syndrome | 0.93 (0.86; 1.02) | 0.11 | 557 |
| Superficial cellulitis and abscess | 1.01 (0.94; 1.09) | 0.70 | 816 |
| Symptoms affecting skin | 1.00 (0.95; 1.06) | 0.87 | 1,246 |
| Systemic lupus erythematosus | 1.02 (0.92; 1.13) | 0.71 | 392 |
| Systemic sclerosis | 1.08 (0.94; 1.24) | 0.31 | 207 |
| Unspecified erythematous condition | 0.94 (0.86; 1.03) | 0.20 | 458 |
| Urticaria | 0.91 (0.82; 1.01) | 0.07 | 377 |
| **Digestive** | | | |
| Abdominal hernia | 1.00 (0.99; 1.01) | 0.68 | 34,106 |
| Abnormal results of function study of liver | 1.01 (0.97; 1.04) | 0.68 | 3,562 |
| Abnormal serum enzyme levels | 0.93 (0.82; 1.07) | 0.33 | 224 |
| Acute appendicitis | 0.96 (0.87; 1.05) | 0.34 | 462 |
| Acute gastritis | 1.00 (0.91; 1.11) | 0.95 | 404 |
| Acute pancreatitis | 0.98 (0.94; 1.03) | 0.41 | 1,998 |
| Anal and rectal conditions | 0.99 (0.97; 1.01) | 0.25 | 14,583 |
| Anal and rectal polyp | 0.99 (0.97; 1.01) | 0.33 | 7,464 |
| Appendiceal conditions | 0.95 (0.89; 1.01) | 0.08 | 1,105 |
| Appendicitis | 0.94 (0.88; 1.01) | 0.08 | 908 |
| Ascites (non malignant) | 1.02 (0.98; 1.07) | 0.33 | 1,824 |
| Atrophic gastritis | 0.99 (0.89; 1.09) | 0.82 | 391 |
| Barrett's esophagus | 1.02 (0.99; 1.05) | 0.30 | 3,898 |
| Blood in stool | 1.01 (0.97; 1.05) | 0.56 | 2,619 |
| Calculus of bile duct | 1.03 (0.92; 1.16) | 0.59 | 303 |
| Celiac disease | 1.00 (0.95; 1.05) | 0.95 | 1,906 |
| Cholangitis | 1.05 (0.97; 1.14) | 0.19 | 655 |
| Cholecystitis without cholelithiasis | 1.00 (0.96; 1.04) | 0.88 | 2,482 |
| Cholelithiasis | 1.00 (0.99; 1.02) | 0.73 | 12,524 |
| Cholelithiasis and cholecystitis | 1.00 (0.99; 1.02) | 0.57 | 14,083 |
| Cholelithiasis with acute cholecystitis | 1.01 (0.96; 1.07) | 0.65 | 1,495 |
| Cholelithiasis with other cholecystitis | 0.99 (0.97; 1.02) | 0.69 | 4,911 |
| Cholesterolosis of gallbladder | 1.06 (0.96; 1.17) | 0.27 | 392 |
| Chronic liver disease and cirrhosis | 1.00 (0.97; 1.03) | 0.84 | 4,168 |
| Chronic pancreatitis | 0.99 (0.90; 1.07) | 0.74 | 553 |
| Chronic periodontitis | 1.01 (0.94; 1.07) | 0.86 | 986 |
| Cyst and pseudocyst of pancreas | 0.95 (0.87; 1.05) | 0.34 | 445 |
| Dental caries | 0.97 (0.93; 1.01) | 0.12 | 2,912 |
| Diaphragmatic hernia | 1.00 (0.98; 1.01) | 0.58 | 27,703 |
| Diseases and other conditions of the tongue | 1.01 (0.95; 1.07) | 0.67 | 1,149 |
| Diseases of esophagus | 1.00 (0.99; 1.01) | 0.77 | 35,697 |
| Diseases of hard tissues of teeth | 0.97 (0.94; 1.01) | 0.15 | 2,970 |
| Diseases of lips | 0.99 (0.91; 1.08) | 0.86 | 595 |
| Diseases of pancreas | 0.98 (0.95; 1.02) | 0.33 | 2,969 |
| Diseases of pulp and periapical tissues | 1.00 (0.95; 1.06) | 0.90 | 1,601 |
| Diseases of the oral soft tissues, excluding lesions specific for gingiva and tongue | 0.98 (0.94; 1.03) | 0.49 | 1,744 |
| Diseases of the salivary glands | 1.04 (0.96; 1.12) | 0.40 | 633 |
| Disorders of esophageal motility | 1.04 (0.96; 1.13) | 0.29 | 664 |
| Disorders of function of stomach | 1.00 (0.98; 1.02) | 0.82 | 10,886 |
| Disorders of tooth development | 1.00 (0.95; 1.04) | 0.83 | 1,986 |
| Disturbances in tooth eruption | 1.00 (0.96; 1.05) | 0.98 | 1,923 |
| Duodenal ulcer | 1.00 (0.97; 1.04) | 0.83 | 2,772 |
| Duodenitis | 1.02 (0.99; 1.04) | 0.22 | 7,019 |
| Dyspepsia and other specified disorders of function of stomach | 1.00 (0.98; 1.02) | 0.83 | 10,878 |
| Esophageal bleeding (varices/hemorrhage) | 1.00 (0.94; 1.06) | 0.94 | 1,114 |
| Esophagitis, GERD and related diseases | 1.00 (0.99; 1.01) | 0.82 | 33,634 |
| Femoral hernia | 1.00 (0.92; 1.09) | 0.97 | 519 |
| Functional digestive disorders | 0.99 (0.98; 1.01) | 0.25 | 21,502 |
| Gastric ulcer | 1.00 (0.97; 1.03) | 0.95 | 4,156 |
| Gastritis and duodenitis | 1.00 (0.98; 1.01) | 0.59 | 27,792 |
| Gastroesophageal laceration-hemorrhage syndrome | 1.05 (0.94; 1.18) | 0.36 | 311 |
| Gastrointestinal complications | 0.98 (0.90; 1.08) | 0.71 | 508 |
| Gastrointestinal hemorrhage | 0.99 (0.98; 1.01) | 0.44 | 20,174 |
| GERD | 1.00 (0.99; 1.02) | 0.58 | 16,022 |
| Gingival and periodontal diseases | 0.99 (0.95; 1.04) | 0.81 | 1,633 |
| Glossitis | 1.00 (0.89; 1.13) | 0.96 | 283 |
| Heartburn | 0.99 (0.95; 1.04) | 0.66 | 1,881 |
| Hematemesis | 0.99 (0.95; 1.04) | 0.66 | 1,929 |
| Hemorrhage from gastrointestinal ulcer | 1.06 (0.98; 1.15) | 0.16 | 638 |
| Hemorrhage of gastrointestinal tract | 1.00 (0.98; 1.03) | 0.87 | 6,196 |
| Hemorrhage of rectum and anus | 1.00 (0.98; 1.01) | 0.64 | 11,448 |
| Hepatomegaly | 1.08 (0.96; 1.21) | 0.21 | 297 |
| Ileostomy status | 0.99 (0.95; 1.05) | 0.84 | 1,524 |
| Impaction of intestine | 1.08 (0.96; 1.22) | 0.17 | 301 |
| Incisional hernia | 1.02 (0.90; 1.15) | 0.78 | 275 |
| Inflammatory bowel disease and other gastroenteritis and colitis | 0.97 (0.95; 1.00) | 0.08 | 4,986 |
| Inguinal hernia | 1.02 (0.91; 1.15) | 0.69 | 310 |
| Intestinal malabsorption (non-celiac) | 0.95 (0.84; 1.08) | 0.45 | 250 |
| Intestinal obstruction without mention of hernia | 1.02 (0.99; 1.05) | 0.28 | 4,105 |
| Irritable Bowel Syndrome | 0.99 (0.96; 1.02) | 0.47 | 5,249 |
| Jaundice (not of newborn) | 1.01 (0.94; 1.08) | 0.74 | 850 |
| Liver abscess and sequelae of chronic liver disease | 0.98 (0.92; 1.04) | 0.46 | 1,213 |
| Noninfectious gastroenteritis | 0.98 (0.97; 1.00) | 0.05 | 13,550 |
| Nonspecific abnormal findings in stool contents | 1.02 (0.97; 1.07) | 0.46 | 1,853 |
| Nonspecific abnormal findings on radiological and other examination of biliary tract | 1.01 (0.91; 1.12) | 0.89 | 358 |
| Obstruction of bile duct | 1.00 (0.94; 1.07) | 0.98 | 977 |
| Other biliary tract disease | 1.00 (0.97; 1.03) | 0.88 | 4,093 |
| Other chronic nonalcoholic liver disease | 1.00 (0.96; 1.04) | 0.98 | 2,915 |
| Other diseases of the teeth and supporting structures | 0.99 (0.95; 1.04) | 0.78 | 2,303 |
| Other disorders of biliary tract | 1.00 (0.94; 1.06) | 1.00 | 1,112 |
| Other disorders of gallbladder | 0.94 (0.89; 1.00) | 0.04 | 1,310 |
| Other disorders of intestine | 0.98 (0.95; 1.01) | 0.13 | 4,173 |
| Other disorders of liver | 1.02 (0.97; 1.07) | 0.38 | 1,692 |
| Other disorders of peritoneum | 0.99 (0.96; 1.02) | 0.54 | 3,425 |
| Other disorders of stomach and duodenum | 0.99 (0.96; 1.03) | 0.70 | 3,418 |
| Other intestinal obstruction | 1.01 (0.97; 1.05) | 0.70 | 2,809 |
| Other specified gastritis | 1.00 (0.98; 1.02) | 0.88 | 8,311 |
| Other symptoms involving abdomen and pelvis | 1.01 (0.96; 1.07) | 0.69 | 1,482 |
| Paralytic ileus | 1.04 (0.95; 1.13) | 0.42 | 568 |
| Peptic ulcer (excl. esophageal) | 1.01 (0.99; 1.04) | 0.26 | 7,286 |
| Peptic ulcer, site unspecified | 1.14 (1.03; 1.26) | 0.01 | 397 |
| Periapical abscess | 1.00 (0.94; 1.07) | 0.89 | 1,073 |
| Periodontitis (acute or chronic) | 1.00 (0.95; 1.07) | 0.88 | 1,126 |
| Peritoneal adhesions (postoperative) (postinfection) | 0.99 (0.96; 1.03) | 0.62 | 3,209 |
| Peritoneal or intestinal adhesions | 0.98 (0.92; 1.05) | 0.62 | 841 |
| Peritonitis and retroperitoneal infections | 0.97 (0.90; 1.03) | 0.33 | 884 |
| Personal history of diseases of digestive system | 0.99 (0.97; 1.01) | 0.31 | 15,165 |
| Portal hypertension | 0.93 (0.86; 1.01) | 0.09 | 618 |
| Primary biliary cirrhosis | 1.04 (0.93; 1.17) | 0.49 | 297 |
| Reflux esophagitis | 0.99 (0.97; 1.02) | 0.61 | 9,951 |
| Regional enteritis | 0.99 (0.95; 1.04) | 0.69 | 1,894 |
| Sialoadenitis | 1.09 (0.97; 1.22) | 0.13 | 323 |
| Sialolithiasis | 0.98 (0.86; 1.10) | 0.70 | 263 |
| Splenomegaly | 0.97 (0.89; 1.07) | 0.58 | 463 |
| Stomatitis and mucositis | 0.94 (0.86; 1.03) | 0.22 | 463 |
| Stomatitis and mucositis (ulcerative) | 0.93 (0.84; 1.02) | 0.14 | 390 |
| Stricture and stenosis of esophagus | 0.98 (0.93; 1.02) | 0.28 | 2,046 |
| Symptoms involving digestive system | 0.99 (0.97; 1.00) | 0.13 | 13,847 |
| Toxic gastroenteritis and colitis | 0.94 (0.82; 1.08) | 0.40 | 220 |
| Ulcer of esophagus | 1.02 (0.99; 1.05) | 0.24 | 4,755 |
| Ulceration of intestine | 0.89 (0.82; 0.96) | 0.00 | 727 |
| Ulceration of the lower GI tract | 0.91 (0.86; 0.97) | 0.00 | 1,085 |
| Ulcerative colitis | 0.97 (0.94; 1.00) | 0.09 | 3,507 |
| Ulcerative colitis (chronic) | 0.94 (0.86; 1.03) | 0.16 | 550 |
| Umbilical hernia | 1.00 (0.96; 1.03) | 0.86 | 3,507 |
| Ventral hernia | 0.99 (0.96; 1.03) | 0.59 | 3,399 |
| **Endocrine and Metabolic** | | | |
| Abnormal glucose | 0.97 (0.90; 1.04) | 0.36 | 718 |
| Acid-base balance disorder | 0.95 (0.90; 1.00) | 0.05 | 1,384 |
| Acidosis | 0.96 (0.91; 1.02) | 0.18 | 1,242 |
| Adrenal hyperfunction | 0.91 (0.79; 1.04) | 0.16 | 213 |
| Adrenal hypofunction | 0.98 (0.86; 1.11) | 0.73 | 263 |
| Amyloidosis | 0.86 (0.74; 0.99) | 0.03 | 201 |
| Anorexia | 1.02 (0.95; 1.09) | 0.67 | 832 |
| Chondrocalcinosis | 1.06 (0.94; 1.18) | 0.35 | 326 |
| Chronic thyroiditis | 0.95 (0.83; 1.09) | 0.46 | 205 |
| Crystal arthropathies | 1.03 (0.93; 1.14) | 0.62 | 368 |
| Diabetes mellitus | 0.98 (0.97; 1.00) | 0.02 | 19,774 |
| Disorders involving the immune mechanism | 1.13 (1.00; 1.27) | 0.05 | 290 |
| Disorders of adrenal glands | 0.98 (0.90; 1.07) | 0.65 | 593 |
| Disorders of bilirubin excretion | 1.11 (0.99; 1.24) | 0.06 | 344 |
| Disorders of calcium/phosphorus metabolism | 1.02 (0.96; 1.07) | 0.53 | 1,420 |
| Disorders of fluid, electrolyte, and acid-base balance | 0.98 (0.95; 1.00) | 0.03 | 8,325 |
| Disorders of lipoid metabolism | 0.99 (0.98; 1.00) | 0.02 | 33,590 |
| Disorders of magnesium metabolism | 1.02 (0.94; 1.10) | 0.67 | 597 |
| Disorders of mineral metabolism | 1.02 (0.99; 1.06) | 0.23 | 2,709 |
| Disorders of parathyroid gland | 1.03 (0.97; 1.10) | 0.37 | 966 |
| Disorders of plasma protein metabolism | 0.97 (0.88; 1.07) | 0.59 | 419 |
| Disorders of protein plasma/amino-acid transport and metabolism | 0.96 (0.88; 1.06) | 0.43 | 464 |
| Disorders of the pituitary gland and its hypothalamic control | 0.93 (0.86; 1.00) | 0.04 | 774 |
| Electrolyte imbalance | 0.97 (0.95; 1.00) | 0.06 | 5,435 |
| Fluid overload | 0.94 (0.87; 1.01) | 0.11 | 742 |
| Glucocorticoid deficiency | 0.98 (0.87; 1.11) | 0.74 | 262 |
| Gout | 1.06 (1.02; 1.11) | 0.00 | 2,195 |
| Gout and other crystal arthropathies | 1.06 (1.02; 1.11) | 0.00 | 2,541 |
| Graves' disease | 1.04 (0.95; 1.14) | 0.43 | 492 |
| Hypercholesterolemia | 0.98 (0.97; 1.00) | 0.01 | 31,138 |
| Hyperlipidemia | 0.99 (0.98; 1.00) | 0.03 | 33,498 |
| Hyperosmolality and/or hypernatremia | 0.93 (0.83; 1.04) | 0.21 | 329 |
| Hyperparathyroidism | 1.04 (0.97; 1.11) | 0.32 | 871 |
| Hyperpotassemia | 1.03 (0.97; 1.09) | 0.38 | 1,254 |
| Hypoglycemia | 0.98 (0.93; 1.04) | 0.58 | 1,177 |
| Hypopotassemia | 0.99 (0.94; 1.04) | 0.65 | 1,779 |
| Hyposmolality and/or hyponatremia | 0.95 (0.91; 0.99) | 0.01 | 2,525 |
| Hypothyroidism | 0.99 (0.98; 1.01) | 0.41 | 13,761 |
| Hypothyroidism NOS | 0.99 (0.97; 1.01) | 0.32 | 13,094 |
| Hypovolemia | 0.99 (0.96; 1.03) | 0.56 | 3,292 |
| Immunity deficiency | 1.10 (0.97; 1.25) | 0.15 | 251 |
| Mineral deficiency NEC | 0.96 (0.86; 1.07) | 0.46 | 338 |
| Nontoxic multinodular goiter | 0.96 (0.89; 1.04) | 0.29 | 664 |
| Nontoxic nodular goiter | 0.95 (0.90; 1.00) | 0.04 | 1,595 |
| Nontoxic uninodular goiter | 0.95 (0.87; 1.05) | 0.32 | 486 |
| **Obesity** | **0.97 (0.95; 0.98)** | **2.5E-04** | **12,195** |
| Other abnormal glucose | 0.97 (0.88; 1.07) | 0.49 | 430 |
| Other disorders of metabolism | 1.00 (0.93; 1.08) | 0.98 | 683 |
| Other disorders of thyroid | 1.00 (0.91; 1.11) | 0.93 | 403 |
| Other immunological findings | 1.02 (0.90; 1.17) | 0.72 | 224 |
| Ovarian dysfunction | 0.96 (0.84; 1.11) | 0.59 | 208 |
| **Overweight, obesity and other hyperalimentation** | **0.97 (0.95; 0.98)** | **1.9E-04** | **12,318** |
| Paraproteinemia | 0.92 (0.82; 1.04) | 0.17 | 314 |
| Pituitary hyperfunction | 0.96 (0.83; 1.11) | 0.59 | 206 |
| Pituitary hypofunction | 0.90 (0.80; 1.01) | 0.07 | 293 |
| Polycystic ovaries | 0.96 (0.83; 1.11) | 0.59 | 202 |
| Protein-calorie malnutrition | 0.97 (0.86; 1.09) | 0.62 | 275 |
| Proteinuria | 1.10 (0.98; 1.24) | 0.12 | 280 |
| Secondary hypothyroidism | 1.01 (0.96; 1.08) | 0.64 | 1,123 |
| Thyroiditis | 0.94 (0.83; 1.06) | 0.33 | 270 |
| Thyrotoxicosis with or without goiter | 1.02 (0.98; 1.07) | 0.36 | 1,9 |
| Type 1 diabetes | 0.99 (0.95; 1.03) | 0.67 | 2,527 |
| Type 1 diabetes with ketoacidosis | 0.92 (0.81; 1.04) | 0.19 | 253 |
| Type 2 diabetes | 0.98 (0.97; 1.00) | 0.02 | 18,896 |
| Type 2 diabetes with ophthalmic manifestations | 0.98 (0.93; 1.04) | 0.57 | 1,233 |
| Unspecified disorder of lipoid metabolism | 0.91 (0.81; 1.03) | 0.16 | 257 |
| Vitamin B-complex deficiencies | 0.98 (0.92; 1.05) | 0.58 | 868 |
| Vitamin D deficiency | 0.95 (0.88; 1.02) | 0.13 | 890 |
| Vitamin deficiency | 0.98 (0.93; 1.03) | 0.35 | 1,734 |
| **Genitourinary** | | | |
| Abnormal findings on mammogram or breast exam | 0.97 (0.92; 1.03) | 0.37 | 1,252 |
| Abscess or ulceration of vulva | 1.00 (0.88; 1.13) | 1.00 | 255 |
| Acute prostatitis | 0.89 (0.78; 1.01) | 0.08 | 228 |
| Acute renal failure | 0.99 (0.96; 1.01) | 0.29 | 7,161 |
| Anatomical abnormatilies of kidney and ureters | 1.07 (0.94; 1.24) | 0.31 | 214 |
| Atrophy of female genital tract | 0.97 (0.88; 1.08) | 0.58 | 384 |
| Balanoposthitis | 0.91 (0.81; 1.04) | 0.17 | 249 |
| Benign mammary dysplasias | 1.02 (0.98; 1.06) | 0.39 | 2,283 |
| Benign neoplasm of breast | 1.04 (0.98; 1.11) | 0.16 | 1,190 |
| Bladder neck obstruction | 0.96 (0.92; 1.01) | 0.13 | 1,758 |
| Breast conditions, congenital or relating to hormones | 1.08 (1.00; 1.16) | 0.04 | 766 |
| Calculus of kidney | 1.02 (0.98; 1.05) | 0.37 | 3,451 |
| Calculus of lower urinary tract | 1.08 (1.01; 1.16) | 0.03 | 810 |
| Calculus of ureter | 1.03 (0.99; 1.07) | 0.18 | 2,227 |
| Cervicitis and endocervicitis | 0.97 (0.91; 1.04) | 0.42 | 995 |
| Chronic cystitis | 0.98 (0.91; 1.05) | 0.48 | 809 |
| Chronic glomerulonephritis, NOS | 0.95 (0.88; 1.03) | 0.25 | 671 |
| Chronic inflammatory pelvic disease | 1.03 (0.93; 1.15) | 0.54 | 373 |
| Chronic interstitial cystitis | 1.02 (0.90; 1.16) | 0.76 | 244 |
| Chronic kidney disease, Stage I or II | 1.02 (0.91; 1.15) | 0.72 | 312 |
| Chronic Kidney Disease, Stage III | 1.00 (0.96; 1.03) | 0.79 | 2,989 |
| Chronic Kidney Disease, Stage IV | 1.00 (0.92; 1.10) | 0.96 | 510 |
| Chronic prostatitis | 0.98 (0.92; 1.05) | 0.60 | 849 |
| Chronic renal failure [CKD] | 1.01 (0.98; 1.03) | 0.62 | 5,972 |
| Cyst of kidney, acquired | 0.99 (0.94; 1.04) | 0.67 | 1,382 |
| Cyst or abscess of Bartholin's gland | 0.98 (0.91; 1.07) | 0.68 | 625 |
| Cystic mastopathy | 1.01 (0.94; 1.09) | 0.70 | 777 |
| Cystitis | 0.97 (0.93; 1.01) | 0.10 | 2,702 |
| Cystitis and urethritis | 0.96 (0.92; 1.00) | 0.04 | 2,808 |
| Disorders of menstruation and other abnormal bleeding from female genital tract | 1.00 (0.99; 1.02) | 0.66 | 15,411 |
| Disorders of penis | 1.00 (0.94; 1.05) | 0.90 | 1,296 |
| Disorders of uterus, NEC | 1.03 (0.99; 1.07) | 0.16 | 2,829 |
| Dysmenorrhea | 1.02 (0.96; 1.07) | 0.55 | 1,448 |
| Dyspareunia | 1.03 (0.97; 1.10) | 0.31 | 959 |
| Dystrophy of female genital tract | 1.04 (0.92; 1.18) | 0.49 | 271 |
| Dysuria | 1.02 (0.96; 1.08) | 0.62 | 1,119 |
| End stage renal disease | 1.00 (0.93; 1.08) | 0.93 | 808 |
| Endometrial hyperplasia | 1.10 (1.03; 1.18) | 0.01 | 821 |
| Endometriosis | 1.01 (0.98; 1.05) | 0.48 | 3,402 |
| Excessive or frequent menstruation | 1.01 (0.99; 1.03) | 0.34 | 8,292 |
| Fibroadenosis of breast | 0.93 (0.83; 1.06) | 0.27 | 264 |
| Fibrosclerosis of breast | 0.90 (0.80; 1.02) | 0.11 | 258 |
| Frequency of urination and polyuria | 0.99 (0.96; 1.03) | 0.72 | 3,507 |
| Functional disorders of bladder | 0.97 (0.92; 1.02) | 0.25 | 1,397 |
| Genital prolapse | 1.01 (0.99; 1.03) | 0.56 | 10,139 |
| Glomerulonephritis | 1.00 (0.93; 1.07) | 0.97 | 886 |
| Hematuria | 0.99 (0.97; 1.00) | 0.12 | 14,008 |
| Hydrocele | 1.04 (0.98; 1.10) | 0.22 | 1,225 |
| Hydronephrosis | 0.99 (0.93; 1.05) | 0.73 | 956 |
| Hyperplasia of prostate | 0.98 (0.96; 1.00) | 0.08 | 12,295 |
| Hypertrophy of breast (Gynecomastia) | 1.07 (0.99; 1.16) | 0.07 | 696 |
| Hypertrophy of female genital organs | 1.01 (0.95; 1.08) | 0.67 | 1,026 |
| Infertility, female | 1.00 (0.94; 1.06) | 0.89 | 1,160 |
| Inflammatory disease of breast | 0.90 (0.83; 0.98) | 0.01 | 603 |
| Inflammatory disease of cervix, vagina, and vulva | 0.97 (0.93; 1.01) | 0.20 | 2,369 |
| Inflammatory diseases of female pelvic organs | 1.00 (0.97; 1.03) | 0.87 | 5,38 |
| Inflammatory diseases of prostate | 0.98 (0.94; 1.02) | 0.37 | 2,025 |
| Inflammatory diseases of uterus, except cervix | 1.01 (0.89; 1.16) | 0.83 | 241 |
| Irregular menstrual bleeding | 0.99 (0.95; 1.02) | 0.42 | 3,105 |
| Irregular menstrual cycle | 1.00 (0.95; 1.05) | 0.98 | 1,585 |
| Irregular menstrual cycle/bleeding | 1.00 (0.98; 1.02) | 0.86 | 13,085 |
| Kidney replaced by transpant | 0.96 (0.87; 1.07) | 0.45 | 384 |
| Lump or mass in breast | 0.98 (0.92; 1.03) | 0.42 | 1,249 |
| Menopausal and postmenopausal disorders | 1.04 (1.01; 1.06) | 0.00 | 8,995 |
| Mucous polyp of cervix | 1.04 (1.00; 1.08) | 0.05 | 2,775 |
| Nephritis and nephropathy with pathological lesion | 1.13 (1.01; 1.26) | 0.04 | 324 |
| Nephritis and nephropathy without mention of glomerulonephritis | 1.13 (1.01; 1.26) | 0.03 | 336 |
| Nephritis; nephrosis; renal sclerosis | 1.01 (0.97; 1.06) | 0.55 | 1,781 |
| Nephrotic syndrome without mention of glomerulonephritis | 1.01 (0.94; 1.09) | 0.70 | 758 |
| Noninflammatory disorders of cervix | 1.02 (0.98; 1.06) | 0.36 | 2,451 |
| Noninflammatory disorders of ovary, fallopian tube, and broad ligament | 1.02 (0.93; 1.10) | 0.72 | 577 |
| Noninflammatory disorders of vagina | 0.99 (0.94; 1.04) | 0.69 | 1,470 |
| Noninflammatory disorders of vulva and perineum | 0.96 (0.91; 1.02) | 0.18 | 1,168 |
| Noninflammatory female genital disorders | 1.01 (0.99; 1.04) | 0.25 | 7,848 |
| Other disorders of bladder | 1.00 (0.98; 1.02) | 0.92 | 9,358 |
| Other disorders of male genital organs | 1.00 (0.96; 1.04) | 0.82 | 2,397 |
| Other disorders of prostate | 1.03 (0.97; 1.09) | 0.42 | 1,122 |
| Other disorders of testis | 1.02 (0.97; 1.08) | 0.46 | 1,316 |
| Other disorders of the kidney and ureters | 1.01 (0.97; 1.04) | 0.72 | 3,486 |
| Other disorders of urethra and urinary tract | 0.99 (0.95; 1.02) | 0.40 | 3,873 |
| Other inflammatory disorders of male genital organs | 1.10 (0.98; 1.24) | 0.12 | 272 |
| Other nonmalignant breast conditions | 0.98 (0.93; 1.03) | 0.41 | 1,626 |
| Other signs and symptoms in breast | 1.03 (0.94; 1.12) | 0.53 | 573 |
| Other specified benign mammary dysplasias | 0.93 (0.86; 1.02) | 0.11 | 573 |
| Other specified disorders of breast | 1.06 (0.94; 1.2) | 0.32 | 283 |
| Other symptoms/disorders or the urinary system | 1.00 (0.98; 1.03) | 0.64 | 9,573 |
| Ovarian cyst | 1.02 (0.99; 1.06) | 0.14 | 4,141 |
| Pain and other symptoms associated with female genital organs | 1.02 (0.98; 1.07) | 0.31 | 1,875 |
| Pelvic inflammatory disease (PID) | 1.02 (0.95; 1.10) | 0.54 | 822 |
| Pelvic inflammatory disease, NOS | 1.03 (0.92; 1.16) | 0.62 | 298 |
| Pelvic peritoneal adhesions, female (postoperative) (postinfection) | 1.02 (0.98; 1.06) | 0.42 | 2,318 |
| Peyronie's disease | 1.05 (0.97; 1.13) | 0.26 | 669 |
| Polyp of corpus uteri | 1.03 (1.01; 1.06) | 0.01 | 6,836 |
| Polyp of female genital organs | 1.03 (1.01; 1.06) | 0.00 | 9,169 |
| Postmenopausal atrophic vaginitis | 0.98 (0.92; 1.04) | 0.53 | 979 |
| **Postmenopausal bleeding** | **1.04 (1.02; 1.07)** | **2.6E-04** | **7,715** |
| Premenopausal menorrhagia | 1.05 (0.93; 1.19) | 0.44 | 266 |
| Prolapse of vaginal vault after hysterectomy | 0.96 (0.87; 1.06) | 0.40 | 412 |
| Prolapse of vaginal walls | 1.00 (0.97; 1.02) | 0.81 | 6,395 |
| Prostatitis | 0.98 (0.93; 1.03) | 0.48 | 1,497 |
| Pyelonephritis | 0.99 (0.93; 1.04) | 0.60 | 1,436 |
| Renal colic | 0.99 (0.94; 1.04) | 0.68 | 1,686 |
| Renal dialysis | 1.02 (0.93; 1.11) | 0.74 | 522 |
| Renal failure | 1.00 (0.98; 1.02) | 0.83 | 12,055 |
| Renal failure NOS | 1.04 (0.99; 1.10) | 0.13 | 1,326 |
| Retention of urine | 0.98 (0.96; 1.00) | 0.07 | 7,862 |
| stress incontinence, female | 1.00 (0.97; 1.03) | 0.83 | 4,947 |
| Stricture/obstruction of ureter | 1.02 (0.96; 1.09) | 0.55 | 954 |
| Symptoms involving female genital tract | 1.04 (0.96; 1.12) | 0.33 | 759 |
| Urethral stricture (not specified as infectious) | 0.98 (0.94; 1.01) | 0.20 | 3,381 |
| Urinary calculus | 1.01 (0.99; 1.04) | 0.27 | 6,396 |
| Urinary incontinence | 1.00 (0.96; 1.04) | 0.91 | 2,429 |
| Urinary tract infection | 0.99 (0.97; 1.01) | 0.38 | 13,621 |
| Uterine/Uterovaginal prolapse | 1.02 (0.99; 1.05) | 0.19 | 4,63 |
| Vaginal enterocele, congenital or acquired | 1.00 (0.92; 1.09) | 0.95 | 583 |
| Vaginitis and vulvovaginitis | 0.97 (0.89; 1.06) | 0.54 | 530 |
| **Hematopoietic** | | | |
| Acute posthemorrhagic anemia | 0.93 (0.82; 1.06) | 0.31 | 238 |
| Anemia in neoplastic disease | 1.00 (0.91; 1.10) | 0.99 | 422 |
| Anemia of chronic disease | 0.96 (0.89; 1.03) | 0.26 | 791 |
| Aplastic anemia | 0.97 (0.89; 1.06) | 0.54 | 531 |
| Coagulation defects | 1.06 (0.99; 1.13) | 0.09 | 977 |
| Congenital coagulation defects | 1.09 (0.99; 1.19) | 0.10 | 438 |
| Congenital deficiency of other clotting factors (including factor VII) | 1.16 (1.02; 1.32) | 0.02 | 260 |
| Decreased white blood cell count | 1.00 (0.97; 1.04) | 0.81 | 3,387 |
| Diseases of spleen | 0.95 (0.87; 1.04) | 0.27 | 499 |
| Diseases of white blood cells | 1.02 (0.92; 1.12) | 0.73 | 411 |
| Disorders of iron metabolism | 1.05 (0.98; 1.12) | 0.18 | 826 |
| Folate-deficiency anemia | 0.92 (0.81; 1.06) | 0.25 | 216 |
| Hereditary hemolytic anemias | 1.00 (0.91; 1.10) | 0.98 | 593 |
| Iron deficiency anemia secondary to blood loss (chronic) | 1.01 (0.92; 1.11) | 0.83 | 499 |
| Iron deficiency anemias | 0.98 (0.96; 1.00) | 0.10 | 9,867 |
| Iron deficiency anemias, unspecified or not due to blood loss | 0.98 (0.96; 1.00) | 0.11 | 9,478 |
| Lymphadenitis | 0.98 (0.94; 1.02) | 0.28 | 2,589 |
| Megaloblastic anemia | 0.99 (0.93; 1.06) | 0.82 | 1,061 |
| Neutropenia | 1.00 (0.97; 1.04) | 0.81 | 3,387 |
| Other and unspecified coagulation defects | 1.09 (0.98; 1.21) | 0.12 | 370 |
| Other anemias | 0.98 (0.96; 1.00) | 0.01 | 13,689 |
| Other deficiency anemia | 0.99 (0.93; 1.05) | 0.70 | 1,131 |
| Other diseases of blood and blood-forming organs | 0.98 (0.92; 1.05) | 0.55 | 962 |
| Other hemoglobinopathies | 1.02 (0.87; 1.19) | 0.85 | 212 |
| Pernicious anemia | 1.03 (0.96; 1.12) | 0.39 | 698 |
| Personal history of diseases of blood and blood-forming organs | 1.01 (0.90; 1.13) | 0.86 | 312 |
| Polycythemia, secondary | 1.07 (0.95; 1.19) | 0.26 | 324 |
| Primary thrombocytopenia | 0.97 (0.89; 1.07) | 0.54 | 478 |
| Purpura and other hemorrhagic conditions | 1.02 (0.97; 1.06) | 0.52 | 1,937 |
| Sickle cell anemia | 1.01 (0.87; 1.18) | 0.86 | 295 |
| Thrombocytopenia | 1.01 (0.97; 1.06) | 0.55 | 1,806 |
| **Infectious diseases** | | | |
| Bacterial enteritis | 1.01 (0.97; 1.05) | 0.58 | 2,653 |
| Bacterial infection NOS | 0.99 (0.97; 1.02) | 0.67 | 7,883 |
| Candidiasis | 0.96 (0.91; 1.01) | 0.15 | 1,535 |
| Chronic hepatitis | 0.96 (0.86; 1.08) | 0.52 | 309 |
| Dermatophytosis | 1.15 (1.01; 1.30) | 0.04 | 243 |
| Dermatophytosis / Dermatomycosis | 1.09 (0.97; 1.23) | 0.15 | 287 |
| E. coli | 1.00 (0.96; 1.03) | 0.84 | 2,809 |
| Gram negative septicemia | 0.93 (0.83; 1.05) | 0.25 | 276 |
| Gram positive septicemia | 0.93 (0.85; 1.01) | 0.08 | 518 |
| Hepatitis NOS | 0.96 (0.85; 1.08) | 0.50 | 263 |
| Herpes zoster | 1.03 (0.94; 1.14) | 0.48 | 449 |
| Infection/inflammation of internal prosthetic device; implant; and graft | 1.00 (0.95; 1.04) | 0.82 | 2,223 |
| Intestinal infection | 1.00 (0.97; 1.03) | 0.97 | 3,864 |
| Intestinal infection due to C. difficile | 1.09 (1.01; 1.18) | 0.02 | 696 |
| Methicillin sensitive Staphylococcus aureus | 1.01 (0.96; 1.05) | 0.82 | 2,079 |
| Mycoses | 0.95 (0.85; 1.07) | 0.41 | 288 |
| Postoperative infection | 1.00 (0.97; 1.03) | 0.82 | 4,125 |
| Septicemia | 1.01 (0.98; 1.04) | 0.55 | 4,947 |
| Staphylococcus infections | 1.00 (0.97; 1.04) | 0.90 | 2,87 |
| Streptococcus infection | 0.99 (0.94; 1.05) | 0.77 | 1,506 |
| Viral Enteritis | 0.98 (0.91; 1.06) | 0.63 | 742 |
| Viral hepatitis | 1.08 (1.00; 1.18) | 0.06 | 640 |
| Viral hepatitis C | 1.11 (0.99; 1.24) | 0.07 | 341 |
| Viral infection | 1.00 (0.95; 1.04) | 0.89 | 2,040 |
| Viral warts & HPV | 1.08 (1.01; 1.14) | 0.02 | 1,073 |
| Viremia, NOS | 0.99 (0.94; 1.05) | 0.83 | 1,495 |
| **Injuries and poisonings** | | | |
| Adverse drug events and drug allergies | 0.88 (0.78; 0.99) | 0.03 | 281 |
| Adverse effects of sedatives or other central nervous system depressants and anesthetics | 0.95 (0.86; 1.05) | 0.35 | 383 |
| Allergy/adverse effect of penicillin | 0.99 (0.97; 1.01) | 0.26 | 15,271 |
| Anaphylactic shock NOS | 0.94 (0.86; 1.03) | 0.18 | 536 |
| Certain early complications of trauma or procedure | 1.00 (0.88; 1.14) | 0.98 | 224 |
| Complication due to other implant and internal device | 1.02 (0.90; 1.15) | 0.76 | 271 |
| Complication of internal orthopedic device | 1.00 (0.95; 1.04) | 0.86 | 1,886 |
| Contusion | 0.99 (0.94; 1.05) | 0.77 | 1,274 |
| Dislocation | 1.01 (0.93; 1.10) | 0.83 | 537 |
| Effects radiation NOS | 0.97 (0.93; 1.00) | 0.04 | 3,799 |
| Fracture of ankle and foot | 0.99 (0.94; 1.04) | 0.71 | 1,358 |
| Fracture of clavicle or scapula | 0.96 (0.90; 1.02) | 0.21 | 960 |
| Fracture of foot | 0.98 (0.90; 1.06) | 0.58 | 605 |
| Fracture of hand or wrist | 1.00 (0.95; 1.05) | 0.91 | 1,676 |
| Fracture of humerus | 0.99 (0.95; 1.05) | 0.84 | 1,532 |
| Fracture of lower limb | 0.98 (0.95; 1.02) | 0.34 | 3,413 |
| Fracture of neck of femur | 0.99 (0.94; 1.04) | 0.72 | 1,725 |
| Fracture of patella | 1.03 (0.93; 1.13) | 0.61 | 401 |
| Fracture of pelvis | 0.95 (0.88; 1.03) | 0.22 | 677 |
| Fracture of radius and ulna | 1.02 (0.99; 1.05) | 0.27 | 5,113 |
| Fracture of ribs | 1.01 (0.90; 1.13) | 0.89 | 286 |
| Fracture of tibia and fibula | 0.99 (0.93; 1.05) | 0.80 | 1,100 |
| Fracture of unspecified bones | 0.90 (0.80; 1.02) | 0.09 | 269 |
| Fracture of unspecified part of femur | 0.92 (0.82; 1.04) | 0.18 | 284 |
| Fracture of upper limb | 1.00 (0.98; 1.03) | 0.74 | 7,349 |
| Injuries to the nervous system | 0.94 (0.87; 1.01) | 0.09 | 728 |
| Internal derangement of knee | 0.99 (0.97; 1.01) | 0.39 | 12,174 |
| Joint/ligament sprain | 1.02 (0.90; 1.16) | 0.71 | 262 |
| Mechanical complication of unspecified genitourinary device, implant, and graft | 0.98 (0.92; 1.05) | 0.56 | 919 |
| Open wounds of extremities | 0.97 (0.90; 1.05) | 0.45 | 746 |
| Open wounds of head; neck; and trunk | 0.97 (0.90; 1.04) | 0.38 | 761 |
| Opiates and related narcotics causing adverse effects in therapeutic use | 0.98 (0.93; 1.04) | 0.46 | 1,268 |
| Other open wound of head and face | 0.94 (0.85; 1.04) | 0.25 | 379 |
| Personal history of allergy to medicinal agents | 0.98 (0.95; 1.01) | 0.15 | 5,574 |
| Poisoning by analgesics, antipyretics, and antirheumatics | 0.98 (0.95; 1.01) | 0.14 | 4,502 |
| Poisoning by antibiotics | 0.99 (0.97; 1.00) | 0.09 | 17,594 |
| Poisoning by other anti-infectives | 0.97 (0.87; 1.08) | 0.60 | 361 |
| Poisoning/allergy of sulfonamides | 0.97 (0.90; 1.04) | 0.37 | 761 |
| Sepsis | 1.02 (0.99; 1.05) | 0.29 | 4,601 |
| Sepsis and SIRS | 1.02 (0.99; 1.05) | 0.29 | 4,601 |
| Skull and face fracture and other intercranial injury | 0.97 (0.91; 1.04) | 0.37 | 900 |
| Sprains and strains | 1.01 (0.92; 1.10) | 0.86 | 500 |
| Traumatic amputation | 1.04 (0.94; 1.16) | 0.43 | 341 |
| **Mental disorders** | | | |
| Adjustment reaction | 0.99 (0.87; 1.12) | 0.83 | 240 |
| Agorophobia, social phobia, and panic disorder | 0.99 (0.92; 1.06) | 0.79 | 813 |
| Alcoholic liver damage | 0.97 (0.91; 1.03) | 0.30 | 932 |
| Alcoholism | 1.01 (0.97; 1.04) | 0.75 | 3,505 |
| Alcohol-related disorders | 0.99 (0.96; 1.02) | 0.54 | 4,856 |
| Alteration of consciousness | 0.98 (0.89; 1.08) | 0.72 | 423 |
| Altered mental status | 1.00 (0.96; 1.04) | 0.86 | 2,628 |
| Alzheimer's disease | 1.02 (0.95; 1.10) | 0.58 | 727 |
| Anxiety disorder | 0.99 (0.97; 1.02) | 0.44 | 6,310 |
| Anxiety disorders | 0.99 (0.97; 1.01) | 0.39 | 6,889 |
| Aphasia/speech disturbance | 0.99 (0.93; 1.05) | 0.71 | 958 |
| Bipolar | 1.01 (0.95; 1.08) | 0.70 | 1,017 |
| Delirium dementia and amnestic and other cognitive disorders | 1.01 (0.97; 1.06) | 0.59 | 1,895 |
| Dementias | 1.00 (0.95; 1.06) | 0.92 | 1,448 |
| Depression | 1.00 (0.99; 1.02) | 0.61 | 12,201 |
| Develomental delays and disorders | 1.13 (1.04; 1.24) | 0.01 | 512 |
| Hallucinations | 1.03 (0.93; 1.15) | 0.57 | 337 |
| Major depressive disorder | 1.00 (0.99; 1.02) | 0.60 | 12,154 |
| Memory loss | 0.99 (0.92; 1.07) | 0.76 | 691 |
| Mood disorders | 1.01 (0.99; 1.02) | 0.54 | 12,807 |
| Neurological disorders | 1.00 (0.97; 1.03) | 0.82 | 4,210 |
| Other mental disorder | 0.97 (0.87; 1.09) | 0.62 | 326 |
| Other specified nonpsychotic and/or transient mental disorders | 0.97 (0.88; 1.06) | 0.51 | 451 |
| Personality disorders | 0.96 (0.87; 1.07) | 0.47 | 365 |
| Phobia | 0.99 (0.91; 1.07) | 0.76 | 626 |
| Psychogenic and somatoform disorders | 0.95 (0.86; 1.05) | 0.28 | 418 |
| Psychogenic disorder | 0.92 (0.82; 1.03) | 0.15 | 300 |
| Schizophrenia | 0.98 (0.90; 1.06) | 0.58 | 668 |
| Schizophrenia and other psychotic disorders | 0.98 (0.91; 1.05) | 0.60 | 815 |
| Senile dementia | 0.99 (0.93; 1.06) | 0.84 | 870 |
| Substance addiction and disorders | 0.97 (0.86; 1.09) | 0.58 | 314 |
| Suicidal ideation or attempt | 0.91 (0.79; 1.04) | 0.18 | 213 |
| Swelling, mass, or lump in head and neck [Space-occupying lesion, intracranial NOS] | 0.95 (0.89; 1.02) | 0.20 | 795 |
| Symptoms involving head and neck | 0.99 (0.95; 1.04) | 0.81 | 1,794 |
| Tension headache | 0.99 (0.88; 1.12) | 0.90 | 283 |
| Tobacco use disorder | 1.00 (0.97; 1.02) | 0.64 | 9,480 |
| **Musculoskeletal** | | | |
| Acquired deformities of finger | 1.14 (0.99; 1.30) | 0.07 | 219 |
| Acquired deformities of knee | 1.01 (0.88; 1.15) | 0.90 | 234 |
| Acquired foot deformities | 1.01 (0.99; 1.03) | 0.39 | 8,822 |
| Acquired spondylolisthesis | 1.00 (0.95; 1.06) | 0.93 | 1,488 |
| Acquired toe deformities | 1.02 (0.99; 1.05) | 0.25 | 4,489 |
| Ankylosing spondylitis | 1.00 (0.90; 1.11) | 0.96 | 378 |
| Articular cartilage disorder | 0.99 (0.89; 1.10) | 0.84 | 343 |
| Aseptic necrosis of bone | 0.93 (0.81; 1.06) | 0.30 | 217 |
| Bursitis | 0.99 (0.91; 1.07) | 0.75 | 581 |
| Calcaneal spur; Exostosis NOS | 1.04 (0.96; 1.12) | 0.37 | 641 |
| Contracture of joint | 1.02 (0.90; 1.15) | 0.74 | 267 |
| Contracture of palmar fascia [Dupuytren's disease] | 0.99 (0.95; 1.03) | 0.54 | 2,328 |
| Curvature of spine | 0.98 (0.90; 1.06) | 0.61 | 622 |
| Cyst of bone | 1.02 (0.91; 1.15) | 0.75 | 289 |
| Derangement of joint, non-traumatic | 0.99 (0.94; 1.04) | 0.62 | 1,536 |
| Disorders of coccyx | 1.03 (0.90; 1.17) | 0.68 | 247 |
| Disorders of muscle, ligament, and fascia | 0.99 (0.87; 1.12) | 0.82 | 254 |
| Disorders of sacrum | 1.05 (0.93; 1.18) | 0.47 | 273 |
| Enthesopathy | 0.99 (0.97; 1.02) | 0.54 | 7,948 |
| Fasciitis | 0.98 (0.95; 1.02) | 0.45 | 2,537 |
| Ganglion and cyst of synovium, tendon, and bursa | 0.98 (0.95; 1.02) | 0.43 | 2,728 |
| Genu valgum or varum (acquired) | 1.05 (0.94; 1.16) | 0.40 | 358 |
| Hallux rigidus | 0.98 (0.92; 1.03) | 0.38 | 1,331 |
| Hallux valgus (Bunion) | 1.01 (0.98; 1.04) | 0.49 | 5,885 |
| Hammer toe (acquired) | 1.02 (0.97; 1.07) | 0.46 | 1,729 |
| Intervertebral disc disorders | 0.95 (0.90; 1.01) | 0.13 | 1,048 |
| Joint effusions | 0.96 (0.91; 1.01) | 0.14 | 1,347 |
| Kyphoscoliosis and scoliosis | 0.97 (0.89; 1.06) | 0.55 | 533 |
| Laxity of ligament or hypermobility syndrome | 0.98 (0.88; 1.08) | 0.66 | 372 |
| Loose body in joint | 1.00 (0.88; 1.13) | 0.96 | 266 |
| **Osteoarthritis; localized** | **0.97 (0.96; 0.98)** | **1.1E-06** | **33,854** |
| **Osteoarthrosis** | **0.97 (0.96; 0.98)** | **1.1E-08** | **40,154** |
| **Osteoarthrosis NOS** | **0.95 (0.93; 0.97)** | **9.7E-08** | **10,518** |
| Osteoarthrosis, generalized | 0.97 (0.95; 1.00) | 0.03 | 6,19 |
| Osteoarthrosis, localized, primary | 0.97 (0.95; 0.99) | 0.01 | 10,775 |
| Osteoarthrosis, localized, secondary | 1.00 (0.94; 1.05) | 0.91 | 1,252 |
| Osteochondropathies | 0.94 (0.85; 1.04) | 0.22 | 421 |
| Osteomyelitis | 1.01 (0.89; 1.15) | 0.85 | 258 |
| Osteomyelitis, periostitis, and other infections involving bone | 1.07 (0.95; 1.2) | 0.29 | 300 |
| Osteoporosis, osteopenia and pathological fracture | 0.99 (0.93; 1.06) | 0.84 | 921 |
| Other acquired deformities of limbs | 1.03 (0.96; 1.10) | 0.45 | 908 |
| Other acquired musculoskeletal deformity | 1.01 (0.96; 1.06) | 0.78 | 1,832 |
| Other allied disorders of spine | 1.00 (0.90; 1.12) | 0.94 | 350 |
| Other and unspecified disc disorder | 0.95 (0.88; 1.02) | 0.13 | 807 |
| Other and unspecified disorders of back | 1.03 (0.92; 1.15) | 0.59 | 343 |
| Other arthropathies | 1.01 (0.92; 1.12) | 0.79 | 391 |
| Other derangement of joint | 1.01 (0.93; 1.10) | 0.79 | 560 |
| Other disorders of bone and cartilage | 0.97 (0.92; 1.03) | 0.40 | 1,151 |
| Other disorders of soft tissues | 1.08 (0.99; 1.18) | 0.08 | 512 |
| Other disorders of synovium, tendon, and bursa | 0.98 (0.96; 1.00) | 0.05 | 8,381 |
| Other inflammatory spondylopathies | 1.00 (0.95; 1.05) | 0.88 | 1,544 |
| Pain in joint | 0.99 (0.96; 1.01) | 0.29 | 6,655 |
| Pathologic fracture | 1.00 (0.92; 1.09) | 0.96 | 617 |
| Pathologic fracture of vertebrae | 1.00 (0.89; 1.12) | 0.98 | 310 |
| Pathological, developmental or recurrent dislocation | 0.96 (0.87; 1.06) | 0.42 | 418 |
| Peripheral enthesopathies and allied syndromes | 0.99 (0.98; 1.01) | 0.57 | 12,910 |
| Polymyalgia Rheumatica | 1.01 (0.96; 1.07) | 0.67 | 1,292 |
| Rheumatism, unspecified and fibrositis | 1.10 (0.97; 1.23) | 0.13 | 300 |
| Rheumatoid arthritis | 0.98 (0.94; 1.02) | 0.26 | 2,952 |
| Rheumatoid arthritis and other inflammatory polyarthropathies | 0.99 (0.95; 1.02) | 0.40 | 3,314 |
| Rupture of synovium | 0.92 (0.83; 1.02) | 0.09 | 382 |
| Rupture of tendon, nontraumatic | 0.98 (0.94; 1.01) | 0.15 | 3,75 |
| Spinal stenosis | 0.99 (0.96; 1.02) | 0.49 | 4,252 |
| Spinal stenosis of lumbar region | 0.99 (0.96; 1.03) | 0.67 | 3,155 |
| Spondylosis and allied disorders | 0.97 (0.93; 1.01) | 0.15 | 2,069 |
| Spondylosis without myelopathy | 0.96 (0.92; 1.01) | 0.14 | 1,591 |
| Stiffness of joint | 1.03 (0.94; 1.14) | 0.51 | 414 |
| Symptoms and disorders of the joints | 0.98 (0.95; 1.02) | 0.36 | 2,993 |
| Synoviopathy | 0.97 (0.89; 1.06) | 0.51 | 492 |
| Synovitis and tenosynovitis | 1.00 (0.95; 1.05) | 0.95 | 1,568 |
| **Neoplasms** | | | |
| Acquired absence of breast | 0.97 (0.94; 1.01) | 0.12 | 3,198 |
| Benign neoplasm of adrenal gland | 1.03 (0.89; 1.18) | 0.72 | 200 |
| Benign neoplasm of bone and articular cartilage | 1.01 (0.89; 1.13) | 0.93 | 273 |
| Benign neoplasm of brain and other parts of nervous system | 0.97 (0.91; 1.04) | 0.39 | 871 |
| Benign neoplasm of brain, cranial nerves, meninges | 0.98 (0.91; 1.05) | 0.54 | 824 |
| Benign neoplasm of colon | 0.99 (0.98; 1.00) | 0.19 | 21,526 |
| Benign neoplasm of lip, oral cavity, and pharynx | 0.97 (0.91; 1.04) | 0.43 | 814 |
| Benign neoplasm of other endocrine glands and related structures | 0.99 (0.93; 1.06) | 0.85 | 988 |
| Benign neoplasm of other parts of digestive system | 1.00 (0.98; 1.03) | 0.87 | 5,897 |
| Benign neoplasm of ovary | 0.98 (0.93; 1.04) | 0.57 | 1,224 |
| Benign neoplasm of parathyroid gland | 1.02 (0.92; 1.13) | 0.76 | 391 |
| Benign neoplasm of pituitary gland and craniopharyngeal duct (pouch) | 0.95 (0.86; 1.06) | 0.35 | 390 |
| Benign neoplasm of respiratory and intrathoracic organs | 1.03 (0.92; 1.15) | 0.61 | 339 |
| Benign neoplasm of skin | 1.02 (1.00; 1.05) | 0.08 | 5,417 |
| Benign neoplasm of thyroid glands | 0.97 (0.85; 1.10) | 0.62 | 233 |
| Benign neoplasm of unspecified sites | 0.99 (0.86; 1.14) | 0.89 | 205 |
| Benign neoplasm of uterus | 1.02 (1.00; 1.04) | 0.04 | 9,655 |
| Breast cancer | 1.00 (0.98; 1.02) | 0.91 | 7,849 |
| Breast cancer [female] | 1.00 (0.98; 1.02) | 0.92 | 7,708 |
| Cancer of bladder | 0.99 (0.95; 1.03) | 0.51 | 2,878 |
| Cancer of bone and connective tissue | 1.02 (0.93; 1.11) | 0.72 | 493 |
| Cancer of brain | 1.03 (0.95; 1.11) | 0.48 | 711 |
| Cancer of brain and nervous system | 1.03 (0.96; 1.11) | 0.46 | 750 |
| Cancer of bronchus; lung | 1.03 (0.99; 1.06) | 0.11 | 3,622 |
| Cancer of connective tissue | 0.99 (0.89; 1.11) | 0.90 | 353 |
| Cancer of esophagus | 0.93 (0.87; 0.98) | 0.01 | 1,128 |
| Cancer of kidney and renal pelvis | 1.03 (0.97; 1.09) | 0.32 | 1,344 |
| Cancer of larynx | 1.01 (0.89; 1.13) | 0.91 | 285 |
| Cancer of larynx, pharynx, nasal cavities | 1.02 (0.95; 1.09) | 0.64 | 766 |
| Cancer of liver and intrahepatic bile duct | 1.01 (0.94; 1.09) | 0.76 | 682 |
| Cancer of mouth | 1.05 (0.98; 1.13) | 0.19 | 732 |
| Cancer of oropharynx | 1.03 (0.92; 1.15) | 0.64 | 317 |
| Cancer of other female genital organs | 1.00 (0.96; 1.04) | 0.90 | 2,786 |
| Cancer of other female genital organs (excluding uterus and ovary) | 0.95 (0.85; 1.07) | 0.40 | 324 |
| Cancer of other lymphoid, histiocytic tissue | 0.96 (0.92; 1.00) | 0.07 | 2,355 |
| Cancer of other male genital organs | 0.99 (0.95; 1.02) | 0.45 | 3,132 |
| Cancer of prostate | 1.01 (0.98; 1.03) | 0.55 | 8,988 |
| Cancer of stomach | 0.93 (0.87; 1.00) | 0.05 | 844 |
| Cancer of tongue | 1.04 (0.93; 1.15) | 0.51 | 352 |
| Cancer of urinary organs (incl. kidney and bladder) | 0.99 (0.96; 1.02) | 0.65 | 4,202 |
| Cancer within the respiratory system | 1.02 (0.99; 1.05) | 0.15 | 4,306 |
| Cancer, suspected or other | 0.99 (0.97; 1.02) | 0.64 | 5,705 |
| Carcinoma in situ of skin | 1.03 (0.96; 1.11) | 0.36 | 740 |
| Cervical cancer | 1.00 (0.95; 1.05) | 0.97 | 1,604 |
| Cervical cancer and dysplasia | 1.00 (0.97; 1.03) | 0.98 | 3,740 |
| Cervical intraepithelial neoplasia [CIN] [Cervical dysplasia] | 1.01 (0.97; 1.05) | 0.69 | 2,223 |
| Chemotherapy | 0.98 (0.96; 1.00) | 0.10 | 8,982 |
| Colon cancer | 0.96 (0.93; 0.99) | 0.01 | 3,838 |
| Colorectal cancer | 0.98 (0.96; 1.01) | 0.16 | 5,742 |
| Hemangioma and lymphangioma, any site | 1.01 (0.96; 1.07) | 0.64 | 1,347 |
| Hodgkin's disease | 0.98 (0.87; 1.10) | 0.71 | 312 |
| Large cell lymphoma | 0.93 (0.87; 1.00) | 0.04 | 912 |
| Leukemia | 0.99 (0.95; 1.03) | 0.57 | 2,696 |
| Lipoma | 1.01 (0.98; 1.04) | 0.41 | 5,524 |
| Lipoma of skin and subcutaneous tissue | 1.02 (0.98; 1.05) | 0.33 | 3,980 |
| Lymphoid leukemia | 1.03 (0.96; 1.11) | 0.39 | 785 |
| Lymphoid leukemia, chronic | 1.04 (0.96; 1.12) | 0.34 | 701 |
| Malignant neoplasm of bladder | 0.99 (0.95; 1.02) | 0.46 | 2,603 |
| Malignant neoplasm of female breast | 1.00 (0.97; 1.02) | 0.81 | 6,698 |
| Malignant neoplasm of gallbladder and extrahepatic bile ducts | 1.02 (0.91; 1.15) | 0.69 | 317 |
| Malignant neoplasm of kidney, except pelvis | 1.03 (0.97; 1.08) | 0.37 | 1,290 |
| Malignant neoplasm of liver, primary | 1.00 (0.89; 1.13) | 0.96 | 301 |
| Malignant neoplasm of other and ill-defined sites within the digestive organs and peritoneum | 1.02 (0.95; 1.09) | 0.56 | 831 |
| Malignant neoplasm of ovary | 1.00 (0.96; 1.04) | 0.97 | 2,385 |
| Malignant neoplasm of ovary and other uterine adnexa | 1.00 (0.96; 1.04) | 0.88 | 2,437 |
| Malignant neoplasm of rectum, rectosigmoid junction, and anus | 1.01 (0.97; 1.05) | 0.79 | 2,559 |
| Malignant neoplasm of retroperitoneum and peritoneum | 1.13 (1.00; 1.28) | 0.06 | 259 |
| Malignant neoplasm of small intestine, including duodenum | 0.95 (0.83; 1.08) | 0.41 | 240 |
| Malignant neoplasm of testis | 0.98 (0.95; 1.02) | 0.32 | 3,007 |
| Malignant neoplasm of unspecified male genital organ | 0.98 (0.95; 1.02) | 0.42 | 2,864 |
| Malignant neoplasm of uterus | 0.99 (0.94; 1.05) | 0.83 | 1,405 |
| Malignant neoplasm, other | 0.99 (0.97; 1.02) | 0.56 | 5,661 |
| Manlignant and unknown neoplasms of brain and nervous system | 1.02 (0.95; 1.09) | 0.55 | 887 |
| Melanomas of skin | 0.99 (0.95; 1.03) | 0.67 | 2,311 |
| Melanomas of skin, dx or hx | 0.99 (0.95; 1.03) | 0.67 | 2,311 |
| Multiple myeloma | 0.94 (0.88; 1.00) | 0.03 | 1,060 |
| Myeloid leukemia | 1.00 (0.93; 1.08) | 0.92 | 727 |
| Myeloid leukemia, acute | 0.97 (0.89; 1.05) | 0.44 | 546 |
| Myeloproliferative disease | 1.03 (0.98; 1.09) | 0.25 | 1,278 |
| Neoplasm of uncertain behavior | 0.99 (0.94; 1.05) | 0.86 | 1,190 |
| Neoplasm of unspecified nature of digestive system | 1.01 (0.95; 1.09) | 0.69 | 830 |
| Nevus, non-neoplastic | 0.99 (0.91; 1.08) | 0.84 | 537 |
| Nodular lymphoma | 1.05 (0.96; 1.15) | 0.29 | 465 |
| Non-Hodgkins lymphoma | 0.96 (0.92; 1.00) | 0.08 | 2,352 |
| Other benign neoplasm of connective and other soft tissue | 0.99 (0.90; 1.08) | 0.77 | 491 |
| Other benign neoplasm of uterus | 0.90 (0.80; 1.01) | 0.09 | 299 |
| Other non-epithelial cancer of skin | 1.00 (0.97; 1.02) | 0.74 | 5,560 |
| Pancreatic cancer | 0.97 (0.92; 1.03) | 0.38 | 1,167 |
| Polycythemia vera | 1.07 (0.97; 1.17) | 0.16 | 472 |
| Radiotherapy | 0.95 (0.91; 1.00) | 0.04 | 2,008 |
| Secondary malignancy of bone | 1.01 (0.97; 1.05) | 0.67 | 3,055 |
| Secondary malignancy of lymph nodes | 0.97 (0.94; 1.00) | 0.03 | 5,537 |
| Secondary malignancy of respiratory organs | 1.00 (0.96; 1.04) | 0.99 | 2,963 |
| Secondary malignant neoplasm | 0.99 (0.97; 1.01) | 0.29 | 11,763 |
| Secondary malignant neoplasm of digestive systems | 1.02 (0.97; 1.07) | 0.46 | 1,910 |
| Secondary malignant neoplasm of liver | 0.99 (0.96; 1.02) | 0.53 | 3,489 |
| Secondary malignant neoplasm of skin | 0.91 (0.80; 1.04) | 0.16 | 228 |
| Skin cancer | 1.00 (0.98; 1.02) | 0.81 | 8,105 |
| Thyroid cancer | 0.92 (0.83; 1.02) | 0.10 | 394 |
| Uterine leiomyoma | 1.03 (1.01; 1.05) | 0.01 | 9,439 |
| Vascular hamartomas and non-neoplastic nevi | 1.01 (0.93; 1.09) | 0.90 | 586 |
| **Neurological** | | | |
| Abnormal involuntary movements | 1.01 (0.94; 1.07) | 0.85 | 961 |
| Abnormal movement | 0.99 (0.94; 1.04) | 0.66 | 1,364 |
| Anterior horn cell disease | 1.08 (0.98; 1.19) | 0.14 | 391 |
| Cerebral degeneration, unspecified | 0.97 (0.88; 1.07) | 0.56 | 407 |
| Cerebral edema and compression of brain | 1.05 (0.93; 1.19) | 0.42 | 276 |
| Coma | 1.01 (0.91; 1.12) | 0.86 | 350 |
| Complex regional/central pain syndrome | 1.00 (0.88; 1.14) | 0.99 | 245 |
| Degenerative disease of the spinal cord | 1.02 (0.97; 1.07) | 0.45 | 1,758 |
| Disorders of other cranial nerves | 0.97 (0.92; 1.03) | 0.32 | 1,395 |
| Disorders of the autonomic nervous system | 0.96 (0.86; 1.07) | 0.47 | 315 |
| Encephalitis | 1.06 (0.94; 1.18) | 0.35 | 309 |
| Epilepsy | 1.04 (0.96; 1.13) | 0.36 | 545 |
| Epilepsy, recurrent seizures, convulsions | 1.00 (0.96; 1.03) | 0.82 | 3,502 |
| Essential tremor | 1.07 (0.95; 1.21) | 0.28 | 269 |
| Extrapyramidal disease and abnormal movement disorders | 1.01 (0.94; 1.08) | 0.88 | 817 |
| Facial nerve disorders [CN7] | 1.01 (0.95; 1.08) | 0.71 | 871 |
| Generalized convulsive epilepsy | 1.03 (0.94; 1.13) | 0.55 | 438 |
| Hemiplegia | 0.99 (0.94; 1.04) | 0.56 | 1,619 |
| Hydrocephalus | 0.97 (0.89; 1.06) | 0.54 | 524 |
| Inflammatory and toxic neuropathy | 1.03 (0.98; 1.09) | 0.23 | 1,418 |
| Lack of coordination | 0.96 (0.87; 1.07) | 0.51 | 338 |
| Meningitis | 0.96 (0.87; 1.06) | 0.42 | 382 |
| Migrain with aura | 1.00 (0.87; 1.15) | 0.95 | 211 |
| Migraine | 1.00 (0.96; 1.04) | 0.94 | 2,916 |
| Multiple sclerosis | 1.02 (0.97; 1.08) | 0.40 | 1,352 |
| Muscular dystrophies and other myopathies | 0.99 (0.88; 1.11) | 0.83 | 313 |
| Myasthenia gravis | 0.91 (0.79; 1.04) | 0.15 | 223 |
| Myoneural disorders | 0.93 (0.82; 1.05) | 0.24 | 253 |
| Nerve root and plexus disorders | 0.95 (0.87; 1.03) | 0.24 | 535 |
| Other and unspecified disorders of the nervous system | 0.96 (0.85; 1.09) | 0.55 | 249 |
| Other cerebral degenerations | 0.96 (0.90; 1.03) | 0.26 | 989 |
| Other CNS infection and poliomyelitis | 1.04 (0.93; 1.16) | 0.53 | 327 |
| Other conditions of brain | 1.03 (0.96; 1.12) | 0.39 | 669 |
| Other conditions of brain, NOS | 1.06 (0.92; 1.22) | 0.45 | 202 |
| Other demyelinating diseases of central nervous system | 1.03 (0.90; 1.17) | 0.69 | 228 |
| Other headache syndromes | 0.99 (0.97; 1.02) | 0.59 | 7,553 |
| Other paralytic syndromes | 0.97 (0.90; 1.06) | 0.53 | 604 |
| Other peripheral nerve disorders | 0.97 (0.95; 0.99) | 0.00 | 11,106 |
| Parkinson's disease | 0.97 (0.93; 1.03) | 0.32 | 1,545 |
| Sleep apnea | 1.01 (0.98; 1.04) | 0.58 | 4,946 |
| Sleep disorders | 0.99 (0.93; 1.05) | 0.63 | 1,160 |
| Torsion dystonia | 0.97 (0.86; 1.09) | 0.60 | 267 |
| Trigeminal nerve disorders [CN5] | 0.92 (0.84; 1.01) | 0.06 | 478 |
| **Pregnancy complications** | | | |
| Antepartum hemorrhage, abruptio placentae, and placenta previa | 0.96 (0.89; 1.04) | 0.30 | 741 |
| Complications of labor and delivery NEC | 0.96 (0.87; 1.06) | 0.45 | 408 |
| Early or threatened labor; hemorrhage in early pregnancy | 1.01 (0.96; 1.05) | 0.77 | 2,178 |
| Ectopic pregnancy | 1.13 (1.00; 1.28) | 0.05 | 273 |
| Excessive vomiting in pregnancy | 0.93 (0.81; 1.06) | 0.27 | 223 |
| Hemorrhage during pregnancy; childbirth and postpartum | 0.98 (0.93; 1.03) | 0.43 | 1,708 |
| Hemorrhage in early pregnancy | 1.01 (0.95; 1.08) | 0.71 | 904 |
| Hypertension complicating pregnancy, childbirth, and the puerperium | 1.00 (0.94; 1.07) | 1.00 | 1,024 |
| Known or suspected fetal abnormality affecting management of mother | 0.94 (0.87; 1.02) | 0.13 | 729 |
| Miscarriage; stillbirth | 1.02 (0.98; 1.06) | 0.36 | 2,477 |
| Missed abortion/Hydatidiform mole | 1.03 (0.96; 1.10) | 0.40 | 999 |
| Other complications of pregnancy NEC | 1.05 (0.97; 1.13) | 0.27 | 669 |
| Placenta previa and abruptio placenta | 0.98 (0.92; 1.05) | 0.62 | 1,083 |
| Preeclampsia and eclampsia | 0.89 (0.79; 1.00) | 0.05 | 299 |
| Problems associated with amniotic cavity and membranes | 1.03 (0.90; 1.18) | 0.68 | 228 |
| **Respiratory** | | | |
| Abnormal findings examination of lungs | 1.02 (0.98; 1.06) | 0.25 | 2,790 |
| Abnormal sputum | 1.00 (0.96; 1.05) | 1.00 | 2,060 |
| Acute and chronic tonsillitis | 1.02 (0.97; 1.07) | 0.48 | 1,738 |
| Acute bronchitis and bronchiolitis | 1.05 (0.92; 1.2) | 0.48 | 217 |
| Acute pharyngitis | 0.99 (0.93; 1.07) | 0.86 | 805 |
| Acute tonsillitis | 0.99 (0.91; 1.08) | 0.85 | 506 |
| Acute upper respiratory infections of multiple or unspecified sites | 1.00 (0.96; 1.04) | 0.99 | 2,184 |
| Allergic rhinitis | 1.05 (0.98; 1.12) | 0.15 | 979 |
| Bacterial pneumonia | 1.00 (0.98; 1.02) | 0.92 | 7,905 |
| Bronchiectasis | 1.00 (0.96; 1.04) | 0.97 | 2,348 |
| Bronchitis | 0.97 (0.89; 1.05) | 0.44 | 571 |
| Bronchopneumonia and lung abscess | 1.02 (0.93; 1.12) | 0.67 | 454 |
| Chronic airway obstruction | 1.00 (0.98; 1.02) | 0.99 | 11,647 |
| Chronic bronchitis | 1.00 (0.97; 1.04) | 0.83 | 3,923 |
| Chronic pharyngitis and nasopharyngitis | 1.09 (1.02; 1.17) | 0.01 | 885 |
| Chronic sinusitis | 0.98 (0.94; 1.02) | 0.40 | 2,291 |
| Chronic tonsillitis and adenoiditis | 1.05 (0.99; 1.12) | 0.11 | 984 |
| Cough | 0.98 (0.94; 1.01) | 0.22 | 2,981 |
| Dependence on respirator [Ventilator] or supplemental oxygen | 1.09 (0.99; 1.2) | 0.09 | 446 |
| Diseases of the larynx and vocal cords | 1.02 (0.98; 1.07) | 0.28 | 2,476 |
| Emphysema | 1.06 (1.02; 1.11) | 0.01 | 1,889 |
| Empyema and pneumothorax | 1.02 (0.96; 1.08) | 0.56 | 1,201 |
| Epistaxis or throat hemorrhage | 1.01 (0.97; 1.05) | 0.62 | 2,305 |
| Hemoptysis | 0.99 (0.95; 1.04) | 0.65 | 1,987 |
| Hyperventilation | 0.98 (0.86; 1.11) | 0.73 | 234 |
| Influenza | 0.95 (0.86; 1.06) | 0.40 | 346 |
| Lung disease due to external agents | 1.03 (0.94; 1.13) | 0.54 | 469 |
| Nasal polyps | 1.02 (0.98; 1.06) | 0.43 | 2,731 |
| Obstructive chronic bronchitis | 1.01 (0.97; 1.04) | 0.76 | 3,663 |
| Other alveolar and parietoalveolar pneumonopathy | 0.97 (0.88; 1.07) | 0.54 | 427 |
| Other diseases of lung | 1.00 (0.94; 1.06) | 0.91 | 998 |
| Other diseases of respiratory system, NEC | 0.99 (0.97; 1.01) | 0.45 | 9,529 |
| Other diseases of respiratory system, not elsewhere classified | 0.99 (0.97; 1.01) | 0.28 | 9,863 |
| Other symptoms of respiratory system | 1.00 (0.98; 1.02) | 0.84 | 8,839 |
| Other upper respiratory disease | 0.99 (0.94; 1.05) | 0.76 | 1,258 |
| Painful respiration | 1.09 (0.95; 1.25) | 0.23 | 216 |
| Paralysis/spasm of vocal cords or larynx | 1.01 (0.89; 1.13) | 0.92 | 297 |
| Pleurisy; pleural effusion | 0.97 (0.95; 1.00) | 0.02 | 7,093 |
| Pneumococcal pneumonia | 1.00 (0.98; 1.03) | 0.90 | 7,405 |
| Pneumoconiosis | 1.02 (0.90; 1.15) | 0.79 | 258 |
| Pneumonia | 1.00 (0.98; 1.02) | 0.76 | 12,015 |
| Pneumonitis due to inhalation of food or vomitus | 1.01 (0.95; 1.08) | 0.70 | 968 |
| Postinflammatory pulmonary fibrosis | 1.03 (0.97; 1.09) | 0.33 | 1,264 |
| Pulmonary collapse; interstitial and compensatory emphysema | 0.98 (0.94; 1.02) | 0.26 | 2,275 |
| Respiratory abnormalities | 0.99 (0.91; 1.08) | 0.81 | 575 |
| Respiratory failure | 1.00 (0.96; 1.04) | 0.90 | 2,488 |
| Respiratory failure, insufficiency, arrest | 1.02 (0.98; 1.05) | 0.39 | 3,046 |
| Respiratory insufficiency | 0.98 (0.94; 1.03) | 0.52 | 1,777 |
| Septal Deviations/Turbinate Hypertrophy | 1.01 (0.98; 1.05) | 0.47 | 3,912 |
| Shortness of breath | 1.01 (0.98; 1.03) | 0.54 | 6,295 |
| Symptoms involving respiratory system and other chest symptoms | 0.97 (0.86; 1.10) | 0.67 | 270 |
| Throat pain | 0.90 (0.81; 1.00) | 0.05 | 342 |
| Voice disturbance | 1.03 (0.96; 1.10) | 0.39 | 976 |
| Wheezing | 0.98 (0.86; 1.12) | 0.79 | 243 |
| **Sense organs** | | | |
| Amblyopia | 1.09 (1.00; 1.19) | 0.05 | 567 |
| Aphakia and other disorders of lens | 1.08 (0.95; 1.23) | 0.23 | 262 |
| Astigmatism | 1.03 (0.90; 1.18) | 0.62 | 223 |
| Blindness and low vision | 0.95 (0.88; 1.02) | 0.15 | 715 |
| Cataract | 0.99 (0.98; 1.01) | 0.41 | 21,932 |
| Cholesteatoma | 1.01 (0.93; 1.11) | 0.76 | 503 |
| Conductive hearing loss | 1.03 (0.93; 1.14) | 0.60 | 375 |
| Conjunctivitis, infectious | 0.94 (0.83; 1.07) | 0.36 | 240 |
| Corneal degenerations | 1.01 (0.89; 1.15) | 0.83 | 244 |
| Corneal dystrophy | 0.94 (0.84; 1.06) | 0.33 | 277 |
| Corneal opacity and other disorders of cornea | 0.97 (0.90; 1.05) | 0.42 | 666 |
| Degeneration of macula and posterior pole of retina | 0.98 (0.94; 1.02) | 0.32 | 2,837 |
| Diplopia and disorders of binocular vision | 0.99 (0.92; 1.07) | 0.82 | 696 |
| Disorders of conjunctiva | 0.94 (0.86; 1.03) | 0.20 | 523 |
| Disorders of external ear | 1.07 (0.98; 1.17) | 0.15 | 492 |
| Disorders of iris and ciliary body | 1.07 (0.94; 1.22) | 0.30 | 246 |
| Disorders of lacrimal system | 1.01 (0.97; 1.06) | 0.59 | 2,156 |
| Disorders of optic nerve and visual pathways | 1.08 (0.97; 1.2) | 0.16 | 364 |
| Disorders of refraction and accommodation; blindness and low vision | 0.99 (0.95; 1.03) | 0.50 | 2,519 |
| Disorders of the globe | 1.04 (0.93; 1.17) | 0.46 | 318 |
| Disorders of vitreous body | 1.04 (0.97; 1.11) | 0.31 | 811 |
| Dizziness and giddiness (Light-headedness and vertigo) | 0.98 (0.96; 1.01) | 0.28 | 4,825 |
| Ectropion or entropion | 0.98 (0.92; 1.05) | 0.58 | 1,052 |
| Epiphora | 1.00 (0.93; 1.07) | 1.00 | 864 |
| Glaucoma | 1.00 (0.97; 1.03) | 0.83 | 5,354 |
| Hearing loss | 1.01 (0.98; 1.04) | 0.53 | 4,545 |
| Hypermetropia | 1.02 (0.90; 1.16) | 0.71 | 251 |
| Impacted cerumen | 1.02 (0.91; 1.15) | 0.74 | 296 |
| Infection of the eye | 1.00 (0.92; 1.09) | 0.98 | 533 |
| Inflammation of eyelids | 0.97 (0.93; 1.02) | 0.24 | 2,009 |
| Inflammation of the eye | 0.97 (0.93; 1.01) | 0.15 | 2,533 |
| Keratitis | 1.08 (0.95; 1.23) | 0.22 | 251 |
| Labyrinthitis | 1.01 (0.94; 1.10) | 0.71 | 674 |
| Macular degeneration (senile) of retina NOS | 0.98 (0.95; 1.02) | 0.35 | 2,834 |
| Meniere's disease | 1.01 (0.93; 1.10) | 0.75 | 555 |
| Myopia | 0.99 (0.93; 1.04) | 0.62 | 1,305 |
| Nystagmus and other irregular eye movements | 1.02 (0.89; 1.17) | 0.79 | 216 |
| Open-angle glaucoma | 1.00 (0.95; 1.05) | 1.00 | 1,485 |
| Otalgia | 0.97 (0.87; 1.10) | 0.67 | 292 |
| Other disorders of eye | 1.03 (0.98; 1.08) | 0.28 | 1,641 |
| Other disorders of eyelids | 0.97 (0.94; 1.00) | 0.07 | 3,665 |
| Other disorders of middle ear and mastoid | 1.02 (0.95; 1.10) | 0.60 | 676 |
| Other disorders of tympanic membrane | 1.00 (0.94; 1.06) | 0.99 | 1,065 |
| Other nondiabetic retinopathy | 1.03 (0.97; 1.10) | 0.33 | 1,021 |
| Other retinal disorders | 0.99 (0.96; 1.03) | 0.72 | 4,207 |
| Otitis externa | 1.02 (0.93; 1.12) | 0.69 | 427 |
| Otitis media | 1.02 (0.97; 1.07) | 0.49 | 1,452 |
| Otitis media and Eustachian tube disorders | 1.01 (0.96; 1.06) | 0.80 | 1,786 |
| Otorrhea | 1.06 (0.93; 1.2) | 0.41 | 235 |
| Otosclerosis | 1.03 (0.91; 1.17) | 0.60 | 268 |
| Paralytic strabismus | 0.89 (0.79; 1.00) | 0.06 | 268 |
| Perforation of tympanic membrane | 0.99 (0.93; 1.07) | 0.85 | 810 |
| Peripheral or central vertigo | 1.05 (0.95; 1.16) | 0.32 | 442 |
| Primary angle-closure glaucoma | 1.00 (0.93; 1.08) | 0.93 | 805 |
| Progressive myopia | 1.07 (0.94; 1.22) | 0.31 | 233 |
| Psychophysical visual disturbances | 1.03 (0.90; 1.18) | 0.67 | 216 |
| Ptosis of eyelid | 0.94 (0.90; 0.99) | 0.01 | 1,791 |
| Retinal detachments and defects | 1.02 (0.95; 1.08) | 0.61 | 994 |
| Senile cataract | 0.99 (0.96; 1.01) | 0.27 | 7,322 |
| Sensorineural hearing loss | 1.01 (0.92; 1.10) | 0.89 | 493 |
| Separation of retinal layers | 1.02 (0.96; 1.09) | 0.48 | 953 |
| Strabismus (not specified as paralytic) | 0.99 (0.92; 1.08) | 0.90 | 606 |
| Strabismus and other disorders of binocular eye movements | 0.98 (0.92; 1.03) | 0.39 | 1,322 |
| Subjective visual disturbances | 1.04 (0.96; 1.13) | 0.31 | 643 |
| Suppurative and unspecified otitis media | 1.04 (0.96; 1.12) | 0.32 | 733 |
| Tinnitus | 1.03 (0.94; 1.12) | 0.54 | 556 |
| Uveitis, noninfectious or NOS | 0.93 (0.84; 1.03) | 0.16 | 406 |
| Vertiginous syndromes and other disorders of vestibular system | 1.02 (0.97; 1.07) | 0.43 | 1,684 |
| Visual disturbances | 1.00 (0.97; 1.04) | 0.91 | 3,015 |
| Visual field defects | 0.97 (0.87; 1.08) | 0.55 | 341 |
| **Symptoms** | | | |
| Abdominal pain | 0.99 (0.97; 1.01) | 0.21 | 16,734 |
| Back pain | 1.00 (0.97; 1.03) | 0.92 | 6,112 |
| Cervicalgia | 0.96 (0.90; 1.02) | 0.20 | 1,033 |
| Chronic fatigue syndrome | 1.00 (0.92; 1.09) | 0.96 | 538 |
| Edema | 0.96 (0.91; 1.00) | 0.06 | 1,858 |
| Fever of unknown origin | 1.00 (0.97; 1.04) | 0.93 | 3,802 |
| Hypothermia/Chills | 0.93 (0.81; 1.07) | 0.32 | 202 |
| Malaise and fatigue | 1.00 (0.96; 1.03) | 0.94 | 3,354 |
| Musculoskeletal symptoms referable to limbs | 1.03 (0.96; 1.11) | 0.42 | 754 |
| Myalgia and myositis unspecified | 1.01 (0.95; 1.08) | 0.69 | 1,092 |
| Nausea and vomiting | 0.98 (0.96; 1.00) | 0.08 | 11,513 |
| Neuralgia, neuritis, and radiculitis NOS | 0.95 (0.86; 1.05) | 0.33 | 402 |
| Other abnormal blood chemistry | 0.94 (0.86; 1.04) | 0.24 | 426 |
| Sciatica | 0.99 (0.93; 1.04) | 0.66 | 1,310 |
| Symptoms involving nervous and musculoskeletal systems | 0.98 (0.95; 1.02) | 0.40 | 3,060 |
| Syncope and collapse | 1.01 (0.99; 1.03) | 0.55 | 9,181 |
| Thoracic or lumbosacral neuritis or radiculitis, unspecified | 1.04 (0.95; 1.14) | 0.40 | 510 |

*Note:* Estimates represent the odds ratio (95% confidence interval) per standard deviation change in instrumented plasma caffeine levels. Bold text indicate statistical significance below a false discovery rate of 5%

**Table S2**. Mendelian randomization estimates for the association of one standard deviation unit increase in genetically predicted plasma caffeine with plasma metabolite levels and ratios.

| **Metabolite** | **MR association estimate** | | **Q statistic**  **p-value** |
| --- | --- | --- | --- |
|  | **Beta (95% CI)** | **p-value** |  |
| **Amino acids** (mmol/L) | | | |
| Alanine | 0.03 (-0.03; 0.09) | 3.1E-01 | 2.3E-01 |
| Glutamine | 0.00 (-0.07; 0.07) | 1.0E+00 | 1.5E-01 |
| **Glycine** | **0.08 (0.03; 0.12)** | **1.1E-03** | **5.1E-01** |
| Histidine | -0.04 (-0.09; 0.01) | 8.8E-02 | 8.2E-01 |
| Isoleucine | -0.01 (-0.06; 0.03) | 5.8E-01 | 7.7E-01 |
| **Leucine** | **-0.06 (-0.10; -0.01)** | **1.8E-02** | **8.6E-01** |
| Phenylalanine | -0.03 (-0.10; 0.04) | 4.6E-01 | 1.4E-01 |
| **Total BCAA** | **-0.06 (-0.11; -0.01)** | **1.1E-02** | **6.0E-01** |
| Tyrosine | -0.06 (-0.10; -0.01) | 2.2E-02 | 4.6E-01 |
| **Valine** | **-0.07 (-0.12; -0.03)** | **1.7E-03** | **4.4E-01** |
| **Apolipoproteins** (g/L) | | | |
| ApoA1 | 0.00 (-0.04; 0.05) | 8.6E-01 | 2.6E-01 |
| **ApoB** | **-0.10 (-0.14; -0.05)** | **9.0E-05** | **9.9E-01** |
| **ApoB/ApoA1** | **-0.08 (-0.13; -0.03)** | **8.0E-04** | **5.8E-01** |
| **Cholesterol** (mmol/L) | | | |
| **Total cholesterol** | **-0.07 (-0.11; -0.02)** | **4.4E-03** | **3.8E-01** |
| **Non-HDL cholesterol** | **-0.09 (-0.14; -0.04)** | **2.5E-04** | **7.6E-01** |
| **Remnant cholesterol** | **-0.10 (-0.15; -0.05)** | **5.6E-05** | **9.4E-01** |
| **VLDL cholesterol** | **-0.12 (-0.17; -0.06)** | **1.4E-05** | **2.7E-01** |
| **LDL cholesterol (clinical)** | **-0.08 (-0.12; -0.03)** | **2.0E-03** | **4.6E-01** |
| **LDL cholesterol (calculated)** | **-0.08 (-0.13; -0.03)** | **1.4E-03** | **6.1E-01** |
| HDL cholesterol | 0.03 (-0.06; 0.12) | 5.2E-01 | 3.5E-02 |
| **Cholesterol in lipoproteins** (mmol/L) | | | |
| **Cholesterol in xs-VLDL** | **-0.10 (-0.15; -0.05)** | **3.3E-05** | **7.8E-01** |
| **Cholesterol in s-VLDL** | **-0.12 (-0.17; -0.07)** | **9.9E-07** | **3.4E-01** |
| **Cholesterol in m-VLDL** | **-0.09 (-0.14; -0.04)** | **1.6E-04** | **9.6E-01** |
| Cholesterol in l-VLDL | -0.11 (-0.2; -0.01) | 3.3E-02 | 3.7E-02 |
| Cholesterol in xl-VLDL | -0.11 (-0.2; -0.01) | 2.3E-02 | 5.1E-02 |
| Cholesterol in CM/xxl-VLDL | -0.09 (-0.2; 0.02) | 1.2E-01 | 1.6E-02 |
| **Cholesterol in s-LDL** | **-0.09 (-0.14; -0.04)** | **3.8E-04** | **9.8E-01** |
| **Cholesterol in m-LDL** | **-0.09 (-0.14; -0.04)** | **1.5E-04** | **7.8E-01** |
| **Cholesterol in l-LDL** | **-0.07 (-0.12; -0.02)** | **4.6E-03** | **3.7E-01** |
| Cholesterol in IDL | -0.06 (-0.13; 0.01) | 8.5E-02 | 1.5E-01 |
| Cholesterol in s-HDL | -0.04 (-0.09; 0.00) | 7.0E-02 | 8.7E-01 |
| Cholesterol in m-HDL | 0.02 (-0.04; 0.08) | 4.5E-01 | 1.7E-01 |
| Cholesterol in l-HDL | 0.04 (-0.07; 0.16) | 4.5E-01 | 6.8E-03 |
| Cholesterol in xl-HDL | 0.04 (-0.07; 0.15) | 5.0E-01 | 9.0E-03 |
| **Cholesterol in lipoproteins ratio** (%) | | | |
| Cholesterol in xs-VLDL | 0.03 (-0.11; 0.17) | 6.8E-01 | 2.2E-03 |
| Cholesterol in s-VLDL | -0.04 (-0.14; 0.07) | 4.9E-01 | 2.9E-02 |
| Cholesterol in m-VLDL | 0.00 (-0.11; 0.11) | 9.9E-01 | 1.4E-02 |
| Cholesterol in l-VLDL | -0.05 (-0.10; 0.00) | 3.5E-02 | 4.2E-01 |
| Cholesterol in xl-VLDL | 0.01 (-0.07; 0.09) | 8.3E-01 | 6.8E-02 |
| Cholesterol in CM/xxl-VLDL | -0.01 (-0.06; 0.04) | 6.1E-01 | 5.4E-01 |
| Cholesterol in s-LDL | -0.05 (-0.10; 0.00) | 3.5E-02 | 8.6E-01 |
| Cholesterol in m-LDL | -0.02 (-0.07; 0.03) | 4.0E-01 | 8.9E-01 |
| Cholesterol in l-LDL | 0.05 (-0.08; 0.18) | 4.2E-01 | 5.8E-03 |
| Cholesterol in IDL | 0.06 (-0.09; 0.20) | 4.7E-01 | 1.4E-03 |
| Cholesterol in s-HDL | 0.06 (-0.05; 0.18) | 2.9E-01 | 1.5E-02 |
| Cholesterol in m-HDL | 0.08 (-0.06; 0.22) | 2.4E-01 | 2.3E-03 |
| Cholesterol in l-HDL | 0.07 (-0.09; 0.23) | 3.9E-01 | 3.3E-04 |
| Cholesterol in xl-HDL | -0.01 (-0.05; 0.04) | 6.9E-01 | 8.2E-01 |
| **Cholesteryl esters in lipoproteins** (mmol/L) | | | |
| **Total** | **-0.06 (-0.11; -0.01)** | **1.2E-02** | **3.1E-01** |
| **Cholesteryl esters in VLDL** | **-0.12 (-0.16; -0.07)** | **2.4E-06** | **4.2E-01** |
| **Cholesteryl esters in xs-VLDL** | **-0.09 (-0.14; -0.04)** | **1.3E-04** | **5.9E-01** |
| **Cholesteryl esters in s-VLDL** | **-0.13 (-0.18; -0.07)** | **8.7E-06** | **2.5E-01** |
| **Cholesteryl esters in m-VLDL** | **-0.08 (-0.13; -0.03)** | **1.1E-03** | **6.8E-01** |
| **Cholesteryl esters in l-VLDL** | **-0.11 (-0.2; -0.02)** | **1.7E-02** | **5.5E-02** |
| **Cholesteryl esters in xl-VLDL** | **-0.11 (-0.2; -0.03)** | **1.1E-02** | **7.2E-02** |
| Cholesteryl esters in CM/xxl-VLDL | -0.09 (-0.2; 0.01) | 8.5E-02 | 2.3E-02 |
| **Cholesteryl esters in LDL** | **-0.08 (-0.13; -0.04)** | **6.1E-04** | **8.0E-01** |
| **Cholesteryl esters in s-LDL** | **-0.09 (-0.14; -0.05)** | **1.1E-04** | **7.2E-01** |
| **Cholesteryl esters in m-LDL** | **-0.10 (-0.15; -0.05)** | **4.5E-05** | **5.1E-01** |
| **Cholesteryl esters in l-LDL** | **-0.07 (-0.12; -0.03)** | **2.8E-03** | **4.6E-01** |
| Cholesteryl esters in IDL | -0.06 (-0.12; 0.01) | 9.6E-02 | 1.5E-01 |
| Cholesteryl esters in HDL | 0.04 (-0.06; 0.13) | 4.6E-01 | 3.2E-02 |
| Cholesteryl esters in s-HDL | -0.04 (-0.08; 0.01) | 1.5E-01 | 9.1E-01 |
| Cholesteryl esters in m-HDL | 0.03 (-0.04; 0.09) | 4.0E-01 | 1.6E-01 |
| Cholesteryl esters in l-HDL | 0.05 (-0.07; 0.16) | 4.2E-01 | 6.6E-03 |
| Cholesteryl esters in xl-HDL | 0.04 (-0.08; 0.15) | 5.1E-01 | 7.5E-03 |
| **Cholesteryl esters in lipoproteins ratio** (%) | | | |
| Cholesteryl esters in xs-VLDL | 0.03 (-0.10; 0.17) | 6.2E-01 | 2.0E-03 |
| Cholesteryl esters in s-VLDL | -0.07 (-0.13; 0.00) | 5.2E-02 | 1.6E-01 |
| Cholesteryl esters in m-VLDL | 0.01 (-0.10; 0.12) | 8.8E-01 | 1.2E-02 |
| Cholesteryl esters in l-VLDL | -0.02 (-0.09; 0.04) | 4.9E-01 | 1.6E-01 |
| Cholesteryl esters in xl-VLDL | 0.01 (-0.07; 0.1) | 7.7E-01 | 6.4E-02 |
| Cholesteryl esters in CM/xxl-VLDL | -0.04 (-0.08; 0.01) | 1.4E-01 | 6.5E-01 |
| Cholesteryl esters in s-LDL | -0.09 (-0.2; 0.01) | 9.0E-02 | 2.2E-02 |
| Cholesteryl esters in m-LDL | -0.10 (-0.24; 0.05) | 1.9E-01 | 2.3E-03 |
| Cholesteryl esters in l-LDL | -0.01 (-0.06; 0.04) | 7.7E-01 | 6.7E-01 |
| Cholesteryl esters in IDL | 0.06 (-0.06; 0.18) | 3.2E-01 | 1.0E-02 |
| Cholesteryl esters in s-HDL | 0.07 (-0.03; 0.16) | 1.7E-01 | 5.0E-02 |
| Cholesteryl esters in m-HDL | 0.09 (-0.04; 0.22) | 1.8E-01 | 4.3E-03 |
| Cholesteryl esters in l-HDL | 0.09 (-0.07; 0.24) | 2.7E-01 | 8.1E-04 |
| Cholesteryl esters in xl-HDL | 0.04 (-0.06; 0.15) | 4.2E-01 | 2.9E-02 |
| **Fatty acids** (mmol/L) | | | |
| **Degree of Unsaturation** | **0.08 (0.04; 0.13)** | **4.9E-04** | **5.6E-01** |
| Docosahexaenoic Acid | 0.02 (-0.03; 0.06) | 5.1E-01 | 4.5E-01 |
| **Linoleic Acid** | **-0.07 (-0.12; -0.02)** | **4.2E-03** | **9.6E-01** |
| **Monounsaturated fatty acids** | **-0.09 (-0.16; -0.02)** | **9.1E-03** | **1.6E-01** |
| Omega-3 | 0.00 (-0.10; 0.09) | 9.3E-01 | 4.9E-02 |
| **Omega-6** | **-0.06 (-0.11; -0.01)** | **1.1E-02** | **7.2E-01** |
| Polyunsaturated fatty acids | -0.05 (-0.10; -0.01) | 2.6E-02 | 4.3E-01 |
| **Saturated fatty acids** | **-0.10 (-0.14; -0.05)** | **6.7E-05** | **4.0E-01** |
| **Total fatty acids** | **-0.09 (-0.14; -0.03)** | **1.1E-03** | **2.8E-01** |
| **Fatty acids** (% of total fatty acids) | | | |
| **Docosahexaenoic Acid** | **0.06 (0.02; 0.11)** | **7.2E-03** | **9.2E-01** |
| Linoleic Acid | 0.03 (-0.06; 0.12) | 4.7E-01 | 5.7E-02 |
| Monounsaturated fatty acids | -0.07 (-0.15; 0.01) | 7.7E-02 | 9.3E-02 |
| Omega-3 | 0.04 (-0.04; 0.13) | 2.9E-01 | 8.4E-02 |
| Omega-6/omega-3 | -0.02 (-0.12; 0.08) | 6.8E-01 | 4.0E-02 |
| Omega-6 | 0.09 (0; 0.17) | 4.4E-02 | 7.1E-02 |
| **Poly-/mono-unsaturated fatty acids** | **0.08 (0.02; 0.15)** | **1.1E-02** | **1.6E-01** |
| **Polyunsaturated fatty acids** | **0.10 (0.05; 0.15)** | **2.1E-05** | **3.6E-01** |
| **Saturated fatty acids** | **-0.09 (-0.14; -0.05)** | **1.1E-04** | **6.8E-01** |
| **Fluid balance** | | | |
| Albumin (g/L) | -0.08 (-0.15; -0.01) | 2.8E-02 | 1.3E-01 |
| **Creatinine (mmol/L)** | **0.09 (0.05; 0.13)** | **2.7E-05** | **7.0E-01** |
| **Free cholesterol in lipoproteins** (mmol/L) | | | |
| **Total** | **-0.08 (-0.13; -0.03)** | **6.3E-04** | **6.2E-01** |
| **Free cholesterol in VLDL** | **-0.11 (-0.18; -0.04)** | **1.2E-03** | **1.5E-01** |
| **Free cholesterol in xs-VLDL** | **-0.11 (-0.16; -0.07)** | **1.7E-06** | **7.4E-01** |
| **Free cholesterol in s-VLDL** | **-0.11 (-0.16; -0.06)** | **1.0E-05** | **5.6E-01** |
| **Free cholesterol in m-VLDL** | **-0.10 (-0.15; -0.05)** | **2.7E-05** | **5.2E-01** |
| Free cholesterol in l-VLDL | -0.10 (-0.2; 0.00) | 5.8E-02 | 2.7E-02 |
| Free cholesterol in xl-VLDL | -0.10 (-0.2; 0.00) | 5.4E-02 | 3.2E-02 |
| Free cholesterol in CM/xxl-VLDL | -0.08 (-0.2; 0.04) | 1.8E-01 | 9.5E-03 |
| Free cholesterol in LDL | -0.06 (-0.12; 0.00) | 3.8E-02 | 2.3E-01 |
| Free cholesterol in s-LDL | -0.06 (-0.11; 0.00) | 3.5E-02 | 2.5E-01 |
| **Free cholesterol in m-LDL** | **-0.07 (-0.12; -0.02)** | **6.0E-03** | **4.2E-01** |
| Free cholesterol in l-LDL | -0.06 (-0.12; 0.01) | 8.3E-02 | 1.8E-01 |
| Free cholesterol in IDL | -0.07 (-0.13; 0.00) | 6.1E-02 | 1.4E-01 |
| Free cholesterol in HDL | 0.01 (-0.07; 0.09) | 7.8E-01 | 6.0E-02 |
| **Free cholesterol in s-HDL** | **-0.06 (-0.11; -0.02)** | **9.5E-03** | **8.6E-01** |
| Free cholesterol in m-HDL | 0.01 (-0.05; 0.06) | 7.4E-01 | 2.1E-01 |
| Free cholesterol in l-HDL | 0.03 (-0.07; 0.14) | 5.4E-01 | 1.1E-02 |
| Free cholesterol in xl-HDL | 0.04 (-0.07; 0.14) | 4.8E-01 | 2.2E-02 |
| **Free cholesterol in lipoproteins ratio** (%) | | | |
| Free cholesterol in xs-VLDL | -0.01 (-0.10; 0.07) | 7.4E-01 | 6.4E-02 |
| Free cholesterol in s-VLDL | 0.01 (-0.12; 0.13) | 8.9E-01 | 6.5E-03 |
| Free cholesterol in m-VLDL | -0.02 (-0.12; 0.08) | 6.6E-01 | 3.0E-02 |
| **Free cholesterol in l-VLDL** | **-0.10 (-0.15; -0.06)** | **2.3E-05** | **4.3E-01** |
| Free cholesterol in xl-VLDL | 0.00 (-0.07; 0.06) | 9.0E-01 | 1.6E-01 |
| Free cholesterol in CM/xxl-VLDL | 0.01 (-0.04; 0.06) | 7.0E-01 | 5.6E-01 |
| Free cholesterol in s-LDL | 0.07 (-0.07; 0.20) | 3.3E-01 | 3.7E-03 |
| Free cholesterol in m-LDL | 0.08 (-0.07; 0.24) | 2.8E-01 | 9.5E-04 |
| Free cholesterol in l-LDL | 0.06 (-0.08; 0.19) | 4.0E-01 | 3.0E-03 |
| Free cholesterol in IDL | 0.01 (-0.08; 0.09) | 9.0E-01 | 6.1E-02 |
| Free cholesterol in s-HDL | 0.00 (-0.10; 0.09) | 9.5E-01 | 3.4E-02 |
| Free cholesterol in m-HDL | 0.02 (-0.08; 0.12) | 7.1E-01 | 1.4E-02 |
| Free cholesterol in l-HDL | -0.01 (-0.13; 0.11) | 8.7E-01 | 8.6E-03 |
| Free cholesterol in xl-HDL | -0.03 (-0.10; 0.04) | 3.9E-01 | 9.2E-02 |
| **Glycolysis related metabolites** (mmol/L) | | | |
| Citrate | 0.04 (-0.10; 0.18) | 5.9E-01 | 2.6E-03 |
| Glucose | -0.04 (-0.09; 0.00) | 7.4E-02 | 6.8E-01 |
| Lactate | -0.02 (-0.07; 0.03) | 4.9E-01 | 4.0E-01 |
| Pyruvate | -0.03 (-0.08; 0.02) | 2.5E-01 | 4.3E-01 |
| **Inflammatory metabolite** (mmol/L) | | | |
| **Glycoprotein Acetyls** | **-0.07 (-0.12; -0.02)** | **4.9E-03** | **5.8E-01** |
| **Ketone bodies** (mmol/L) | | | |
| **3-Hydroxybutyrate** | **-0.07 (-0.12; -0.03)** | **2.6E-03** | **3.9E-01** |
| Acetate | -0.01 (-0.06; 0.04) | 6.3E-01 | 7.8E-01 |
| **Acetoacetate** | **-0.09 (-0.13; -0.04)** | **4.7E-04** | **8.1E-01** |
| Acetone | -0.04 (-0.09; 0.00) | 6.9E-02 | 7.9E-01 |
| **Total lipids in lipoproteins** (mmol/L) | | | |
| **Total** | **-0.09 (-0.14; -0.04)** | **2.6E-04** | **9.0E-01** |
| Total lipids in VLDL | -0.10 (-0.19; -0.01) | 2.2E-02 | 6.0E-02 |
| **Total lipids in xs-VLDL** | **-0.12 (-0.16; -0.07)** | **1.0E-06** | **5.5E-01** |
| **Total lipids in s-VLDL** | **-0.11 (-0.2; -0.03)** | **8.8E-03** | **7.4E-02** |
| **Total lipids in m-VLDL** | **-0.10 (-0.17; -0.03)** | **2.9E-03** | **1.6E-01** |
| Total lipids in l-VLDL | -0.09 (-0.19; 0.01) | 9.2E-02 | 2.8E-02 |
| Total lipids in xl-VLDL | -0.09 (-0.19; 0.01) | 8.5E-02 | 2.9E-02 |
| Total lipids in CM/xxl-VLDL | -0.07 (-0.17; 0.03) | 1.9E-01 | 2.4E-02 |
| **Total lipids in LDL** | **-0.08 (-0.13; -0.04)** | **6.8E-04** | **6.9E-01** |
| **Total lipids in s-LDL** | **-0.09 (-0.13; -0.04)** | **4.3E-04** | **9.5E-01** |
| **Total lipids in m-LDL** | **-0.09 (-0.14; -0.05)** | **1.2E-04** | **7.8E-01** |
| **Total lipids in l-LDL** | **-0.08 (-0.12; -0.03)** | **1.8E-03** | **4.5E-01** |
| **Total lipids in IDL** | **-0.07 (-0.12; -0.02)** | **7.3E-03** | **2.6E-01** |
| Total lipids in HDL | 0.01 (-0.05; 0.07) | 6.9E-01 | 1.6E-01 |
| **Total lipids in s-HDL** | **-0.06 (-0.11; -0.02)** | **8.0E-03** | **3.7E-01** |
| Total lipids in m-HDL | 0.00 (-0.04; 0.05) | 8.7E-01 | 4.7E-01 |
| Total lipids in l-HDL | 0.04 (-0.06; 0.13) | 4.7E-01 | 1.9E-02 |
| Total lipids in xl-HDL | 0.03 (-0.06; 0.13) | 4.9E-01 | 2.2E-02 |
| **Lipoprotein particle concentration** (mmol/L) | | | |
| Total | -0.02 (-0.07; 0.02) | 3.4E-01 | 4.0E-01 |
| **VLDL particle concentration** | **-0.12 (-0.18; -0.05)** | **5.8E-04** | **1.6E-01** |
| **xs-VLDL particle concentration** | **-0.12 (-0.17; -0.07)** | **8.3E-07** | **6.6E-01** |
| **s-VLDL particle concentration** | **-0.11 (-0.2; -0.02)** | **1.4E-02** | **5.7E-02** |
| **m-VLDL particle concentration** | **-0.10 (-0.16; -0.05)** | **6.9E-05** | **2.8E-01** |
| l-VLDL particle concentration | -0.09 (-0.19; 0.01) | 7.9E-02 | 2.7E-02 |
| xl-VLDL particle concentration | -0.09 (-0.19; 0.01) | 8.8E-02 | 2.4E-02 |
| CM/xxl-VLDL particle concentration | -0.08 (-0.19; 0.03) | 1.6E-01 | 1.9E-02 |
| **LDL particle concentration** | **-0.09 (-0.14; -0.04)** | **1.9E-04** | **9.9E-01** |
| **s-LDL particle concentration** | **-0.10 (-0.15; -0.05)** | **4.5E-05** | **9.6E-01** |
| **m-LDL particle concentration** | **-0.10 (-0.15; -0.05)** | **2.8E-05** | **5.8E-01** |
| **l-LDL particle concentration** | **-0.08 (-0.13; -0.03)** | **9.2E-04** | **8.0E-01** |
| **IDL particle concentration** | **-0.08 (-0.13; -0.04)** | **6.2E-04** | **4.2E-01** |
| HDL particle concentration | -0.01 (-0.05; 0.04) | 7.5E-01 | 3.6E-01 |
| s-HDL particle concentration | -0.06 (-0.10; -0.01) | 2.2E-02 | 6.8E-01 |
| m-HDL particle concentration | 0.01 (-0.03; 0.05) | 6.8E-01 | 3.2E-01 |
| l-HDL particle concentration | 0.04 (-0.07; 0.14) | 5.0E-01 | 1.6E-02 |
| xl-HDL particle concentration | 0.03 (-0.07; 0.12) | 6.1E-01 | 2.1E-02 |
| **Lipoprotein particle sizes** (nm) | | | |
| VLDL | -0.05 (-0.15; 0.05) | 3.1E-01 | 2.4E-02 |
| LDL | 0.04 (-0.01; 0.09) | 1.2E-01 | 2.6E-01 |
| HDL | 0.04 (-0.05; 0.14) | 4.1E-01 | 2.5E-02 |
| **Other lipids** (mmol/L) | | | |
| **Total Cholines** | **-0.06 (-0.10; -0.01)** | **1.2E-02** | **7.9E-01** |
| **Phosphatidylcholines** | **-0.06 (-0.10; -0.01)** | **1.3E-02** | **8.4E-01** |
| **Phosphoglycerides** | **-0.06 (-0.11; -0.01)** | **9.3E-03** | **9.7E-01** |
| Sphingomyelins | -0.05 (-0.12; 0.03) | 2.2E-01 | 1.0E-01 |
| **Other lipid ratios** (%) | | | |
| Triglycerides/phosphoglycerides | -0.07 (-0.19; 0.05) | 2.6E-01 | 7.7E-03 |
| **Phospholipids in lipoproteins** (mmol/L) | | | |
| **Total** | **-0.07 (-0.12; -0.02)** | **2.6E-03** | **8.0E-01** |
| **Phospholipids in VLDL** | **-0.11 (-0.19; -0.03)** | **5.6E-03** | **9.6E-02** |
| **Phospholipids in xs-VLDL** | **-0.12 (-0.17; -0.07)** | **7.9E-07** | **4.0E-01** |
| **Phospholipids in s-VLDL** | **-0.11 (-0.17; -0.06)** | **3.2E-05** | **2.7E-01** |
| **Phospholipids in m-VLDL** | **-0.11 (-0.15; -0.06)** | **1.7E-05** | **3.5E-01** |
| Phospholipids in l-VLDL | -0.10 (-0.2; 0.01) | 7.5E-02 | 2.1E-02 |
| Phospholipids in xl-VLDL | -0.10 (-0.2; 0.01) | 6.3E-02 | 3.1E-02 |
| Phospholipids in CM/xxl-VLDL | -0.08 (-0.19; 0.03) | 1.7E-01 | 1.6E-02 |
| **Phospholipids in LDL** | **-0.08 (-0.13; -0.03)** | **8.0E-04** | **6.2E-01** |
| **Phospholipids in s-LDL** | **-0.07 (-0.12; -0.02)** | **3.9E-03** | **5.4E-01** |
| **Phospholipids in m-LDL** | **-0.09 (-0.14; -0.04)** | **3.4E-04** | **9.7E-01** |
| **Phospholipids in l-LDL** | **-0.08 (-0.13; -0.03)** | **1.1E-03** | **4.9E-01** |
| **Phospholipids in IDL** | **-0.08 (-0.12; -0.03)** | **8.6E-04** | **3.8E-01** |
| Phospholipids in HDL | 0.01 (-0.04; 0.05) | 7.8E-01 | 2.9E-01 |
| **Phospholipids in s-HDL** | **-0.06 (-0.11; -0.01)** | **1.0E-02** | **3.2E-01** |
| Phospholipids in m-HDL | 0.00 (-0.05; 0.04) | 8.6E-01 | 6.8E-01 |
| Phospholipids in l-HDL | 0.03 (-0.05; 0.12) | 4.5E-01 | 3.7E-02 |
| Phospholipids in xl-HDL | 0.04 (-0.05; 0.13) | 4.2E-01 | 3.8E-02 |
| **Phospholipids in lipoproteins ratio** (%) | | | |
| Phospholipids in xs-VLDL | -0.03 (-0.12; 0.06) | 5.3E-01 | 4.4E-02 |
| Phospholipids in s-VLDL | 0.01 (-0.12; 0.14) | 8.4E-01 | 5.0E-03 |
| Phospholipids in m-VLDL | -0.04 (-0.14; 0.05) | 3.9E-01 | 4.1E-02 |
| Phospholipids in l-VLDL | -0.11 (-0.22; 0.00) | 4.8E-02 | 2.0E-02 |
| **Phospholipids in xl-VLDL** | **-0.09 (-0.14; -0.04)** | **3.8E-04** | **5.0E-01** |
| Phospholipids in CM/xxl-VLDL | -0.04 (-0.14; 0.07) | 4.9E-01 | 3.2E-02 |
| Phospholipids in s-LDL | 0.09 (-0.01; 0.19) | 6.6E-02 | 3.7E-02 |
| Phospholipids in m-LDL | 0.08 (-0.03; 0.18) | 1.7E-01 | 2.4E-02 |
| Phospholipids in l-LDL | -0.01 (-0.05; 0.04) | 8.0E-01 | 4.1E-01 |
| Phospholipids in IDL | -0.03 (-0.11; 0.05) | 4.5E-01 | 8.9E-02 |
| Phospholipids in s-HDL | 0.00 (-0.04; 0.05) | 8.4E-01 | 4.7E-01 |
| Phospholipids in m-HDL | -0.06 (-0.19; 0.07) | 3.4E-01 | 4.0E-03 |
| Phospholipids in l-HDL | -0.05 (-0.17; 0.08) | 4.6E-01 | 9.3E-03 |
| Phospholipids in xl-HDL | 0.05 (-0.01; 0.11) | 8.0E-02 | 1.9E-01 |
| **Triglycerides in lipoproteins** (mmol/L) | | | |
| Total | -0.09 (-0.2; 0.02) | 1.1E-01 | 2.1E-02 |
| Triglycerides in VLDL | -0.08 (-0.19; 0.03) | 1.4E-01 | 2.2E-02 |
| Triglycerides in xs-VLDL | -0.12 (-0.23; 0.00) | 5.6E-02 | 1.2E-02 |
| Triglycerides in s-VLDL | -0.08 (-0.21; 0.04) | 2.0E-01 | 6.8E-03 |
| Triglycerides in m-VLDL | -0.08 (-0.19; 0.02) | 1.1E-01 | 2.8E-02 |
| Triglycerides in l-VLDL | -0.07 (-0.17; 0.02) | 1.5E-01 | 3.7E-02 |
| Triglycerides in xl-VLDL | -0.08 (-0.18; 0.03) | 1.4E-01 | 2.4E-02 |
| Triglycerides in CM/xxl-VLDL | -0.06 (-0.14; 0.02) | 1.5E-01 | 7.5E-02 |
| **Triglycerides in LDL** | **-0.11 (-0.19; -0.03)** | **6.0E-03** | **8.8E-02** |
| Triglycerides in s-LDL | -0.10 (-0.19; 0.00) | 5.2E-02 | 3.9E-02 |
| **Triglycerides in m-LDL** | **-0.11 (-0.19; -0.02)** | **1.2E-02** | **7.7E-02** |
| **Triglycerides in l-LDL** | **-0.12 (-0.19; -0.04)** | **1.9E-03** | **1.2E-01** |
| **Triglycerides in IDL** | **-0.12 (-0.21; -0.03)** | **1.1E-02** | **4.9E-02** |
| Triglycerides in HDL | -0.10 (-0.21; 0.02) | 9.2E-02 | 1.7E-02 |
| Triglycerides in s-HDL | -0.10 (-0.23; 0.02) | 1.1E-01 | 5.4E-03 |
| Triglycerides in m-HDL | -0.10 (-0.21; 0.02) | 1.0E-01 | 1.4E-02 |
| Triglycerides in l-HDL | -0.06 (-0.11; 0.00) | 4.0E-02 | 2.5E-01 |
| **Triglycerides in xl-HDL** | **-0.07 (-0.13; -0.02)** | **1.0E-02** | **2.4E-01** |
| **Triglycerides in lipoproteins ratio** (%) | | | |
| Triglycerides in xs-VLDL | -0.03 (-0.16; 0.11) | 7.1E-01 | 2.8E-03 |
| Triglycerides in s-VLDL | 0.02 (-0.09; 0.13) | 7.1E-01 | 1.5E-02 |
| Triglycerides in m-VLDL | 0.01 (-0.10; 0.12) | 8.7E-01 | 1.6E-02 |
| **Triglycerides in l-VLDL** | **0.09 (0.05; 0.14)** | **1.0E-04** | **6.7E-01** |
| Triglycerides in xl-VLDL | 0.01 (-0.06; 0.08) | 7.7E-01 | 1.2E-01 |
| Triglycerides in CM/xxl-VLDL | 0.02 (-0.02; 0.07) | 3.2E-01 | 4.1E-01 |
| Triglycerides in s-LDL | -0.05 (-0.18; 0.08) | 4.8E-01 | 4.8E-03 |
| Triglycerides in m-LDL | -0.03 (-0.13; 0.07) | 5.3E-01 | 3.1E-02 |
| Triglycerides in l-LDL | -0.06 (-0.18; 0.07) | 3.9E-01 | 6.6E-03 |
| Triglycerides in IDL | -0.06 (-0.21; 0.09) | 4.5E-01 | 1.3E-03 |
| Triglycerides in s-HDL | -0.09 (-0.22; 0.04) | 1.8E-01 | 4.5E-03 |
| Triglycerides in m-HDL | -0.09 (-0.23; 0.04) | 1.9E-01 | 2.9E-03 |
| Triglycerides in l-HDL | -0.08 (-0.23; 0.06) | 2.7E-01 | 1.1E-03 |
| Triglycerides in xl-HDL | -0.09 (-0.22; 0.04) | 2.0E-01 | 4.1E-03 |

Note: Bold text indicate statistical significance below a false discovery rate of 5%

Abbreviations: Apo: apolipoprotein, CI: confidence interval, CM/xxl-VLDL: chylomicrons and extremely large VLDL, HDL: high-density lipoprotein, IDL: intermediate-density lipoprotein, l: large, LDL: low-density lipoprotein, m: medium, s: small, VLDL: very-low density lipoprotein, XL: very large, XS: very small.
